# EP4 Receptor–Associated Protein in Macrophages Ameliorates Colitis and Colitis-Associated Tumorigenesis

**DOI:** 10.1371/journal.pgen.1005542

**Published:** 2015-10-06

**Authors:** Masato Nakatsuji, Manabu Minami, Hiroshi Seno, Mika Yasui, Hideyuki Komekado, Sei Higuchi, Risako Fujikawa, Yuki Nakanishi, Akihisa Fukuda, Kenji Kawada, Yoshiharu Sakai, Toru Kita, Peter Libby, Hiroki Ikeuchi, Masayuki Yokode, Tsutomu Chiba

**Affiliations:** 1 Department of Gastroenterology and Hepatology, Kyoto University Graduate School of Medicine, Kyoto, Japan; 2 Department of Clinical Innovative Medicine, Kyoto University Graduate School of Medicine, Kyoto, Japan; 3 Department of Surgery, Kyoto University Graduate School of Medicine, Kyoto, Japan; 4 Kobe City Municipal Center General Hospital, Kobe, Japan; 5 Division of Cardiovascular Medicine, Brigham and Women’s Hospital, Harvard Medical School, Boston, Massachusetts, United States of America; 6 Department of Surgery, Hyogo College of Medicine, Nishinomiya, Japan; Seattle Children's Research Institute, UNITED STATES

## Abstract

Prostaglandin E_2_ plays important roles in the maintenance of colonic homeostasis. The recently identified prostaglandin E receptor (EP) 4–associated protein (EPRAP) is essential for an anti-inflammatory function of EP4 signaling in macrophages in vitro. To investigate the *in vivo* roles of EPRAP, we examined the effects of EPRAP on colitis and colitis-associated tumorigenesis. In mice, EPRAP deficiency exacerbated colitis induced by dextran sodium sulfate (DSS) treatment. Wild-type (WT) or EPRAP-deficient recipients transplanted with EPRAP-deficient bone marrow developed more severe DSS-induced colitis than WT or EPRAP-deficient recipients of WT bone marrow. In the context of colitis-associated tumorigenesis, both systemic EPRAP null mutation and EPRAP-deficiency in the bone marrow enhanced intestinal polyp formation induced by azoxymethane (AOM)/DSS treatment. Administration of an EP4-selective agonist, ONO-AE1-329, ameliorated DSS-induced colitis in WT, but not in EPRAP-deficient mice. EPRAP deficiency increased the levels of the phosphorylated forms of p105, MEK, and ERK, resulting in activation of stromal macrophages in DSS-induced colitis. Macrophages of DSS-treated EPRAP-deficient mice exhibited a marked increase in the expression of pro-inflammatory genes, relative to WT mice. By contrast, forced expression of EPRAP in macrophages ameliorated DSS-induced colitis and AOM/DSS-induced intestinal polyp formation. These data suggest that EPRAP in macrophages functions crucially in suppressing colonic inflammation. Consistently, EPRAP-positive macrophages were also accumulated in the colonic stroma of ulcerative colitis patients. Thus, EPRAP may be a potential therapeutic target for inflammatory bowel disease and associated intestinal tumorigenesis.

## Author Summary

Inflammatory bowel disease (IBD) is one of the most prevalent and serious gastrointestinal diseases in Western countries and associates with cancer development. EP4 receptor signaling can suppress intestinal inflammation and shows promise as a target for the development of novel therapies for IBD. To date, however, the lack of detailed molecular targets has hampered the development of effective drugs. This study focused on EPRAP, a novel EP4 receptor–associated protein, implicated in its signaling pathway. The generation of EPRAP-gene mutated mice permitted exploration of EPRAP functions *in vivo*. In addition, EPRAP was localized in stromal macrophages of ulcerative colitis patients. This study revealed that EPRAP in macrophage participates critically in EP4 receptor signaling-mediated inhibition of intestinal inflammation. The macrophage EP4–EPRAP axis thus comprises a novel therapeutic target for IBD.

## Introduction

The incidence of inflammatory bowel disease (IBD) is increasing, and current concepts attribute IBD to inappropriate chronic inflammatory responses to commensal microbes in genetically susceptible patients [1]. Prostaglandin E_2_ (PGE_2_) plays a pivotal role in maintaining local homeostasis in a variety of pathophysiological settings. PGE_2_ receptors (EPs) mediate the effects of this molecule and include four subtypes: EP1–4 [2]. PGE_2_ participates decisively in the defense of the colonic mucosa. For example, misoprostol, a synthetic PGE_1_ analog, potently protects human colonic mucosa against mucosal insults [3]. In addition, in mice, PGE_2_ and EP4-selective agonists significantly improved colitis induced by dextran sodium sulfate (DSS) treatment [4,5]. Yet, misoprostol frequently induces severe diarrhea in humans [6], and treatment with EP receptor agonists can induce vasodilatation, causing hypotension [7]. Indeed, in a phase 2 clinical trial of an EP4-selective agonist for ulcerative colitis patients, diarrhea or hypotension occurred in patients with EP4 agonist treatment [8]. These undesired effects of PGE_2_ and EP4 agonists likely result from increased cAMP production in colonic or vascular endothelial cells. Hence the need is urgent to develop molecules that stimulate EP4 receptors but have fewer side effects to treat IBD, and potentially other inflammatory diseases.

EPRAP, a cytoplasmic EP4–interacting molecule, emerged from yeast two-hybrid screening, using the C-terminus of EP4 receptor as a bait [9]. EPRAP contains multiple ankyrin repeat motifs, but has no predicted enzymatic or catalytic domain. EPRAP transcripts abound in the heart, skeletal muscle, and the kidney, and localize at lower amounts in several other human tissues [10]. The counterpart of EPRAP in mice is Fem1a, a mammalian ortholog of *Caenorhabditis elegans* FEM-1. FEM-1 participates in nematode sex determination [11], although the functions of mouse EPRAP/Fem1a and of human EPRAP remain uncertain.

*In vitro*, EPRAP in macrophages mediates an anti-inflammatory function of PGE_2_–EP4 signaling [12]; however, the pathophysiological roles of EPRAP in colonic inflammation *in vivo* remain unknown. In this study, we evaluated the role of EPRAP in the development of colitis and colitis-associated tumorigenesis using EPRAP-deficient mice and macrophage-specific EPRAP-overexpressing mice.

## Results

### EPRAP deficiency exacerbates DSS-induced colitis

**Fig 1:**
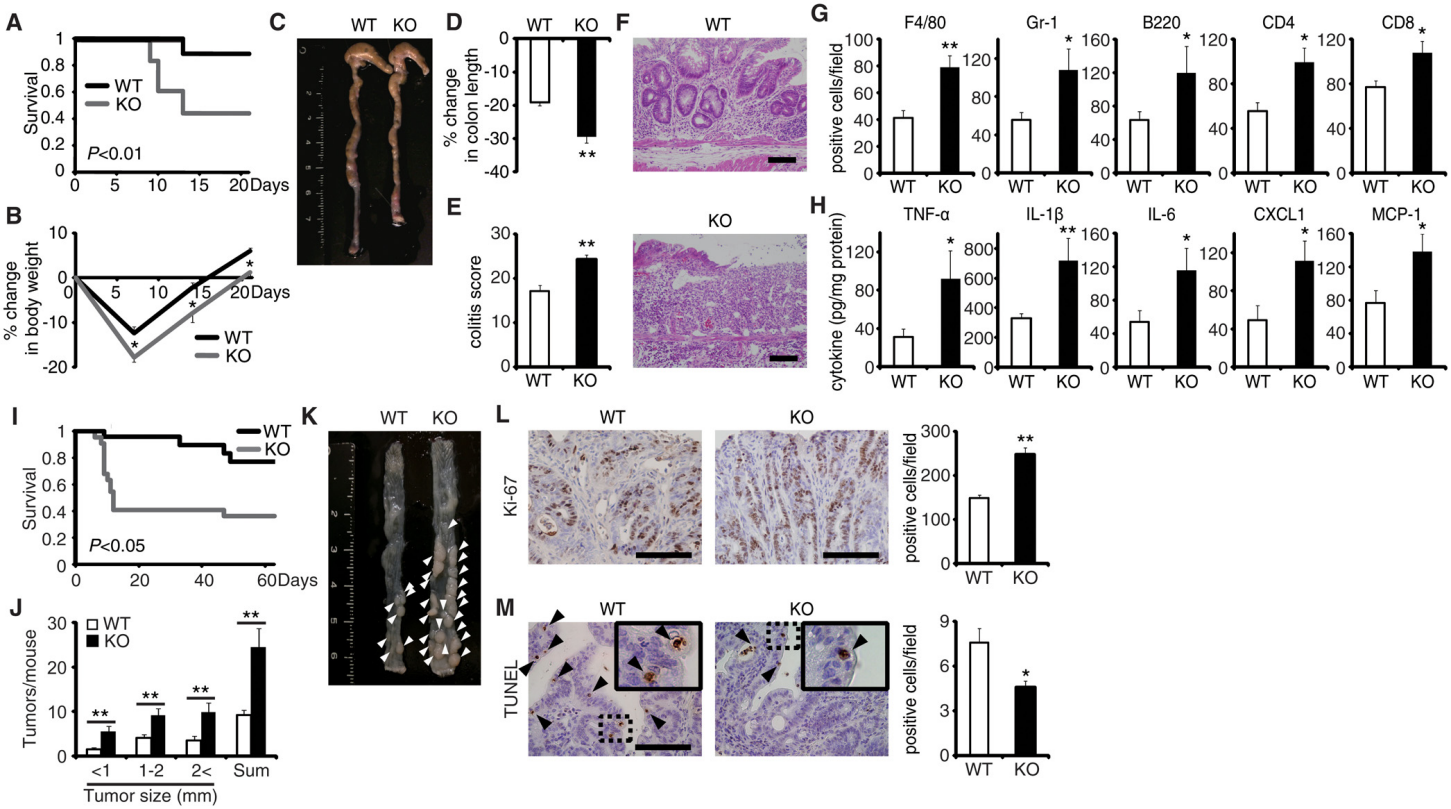
EPRAP deficiency exacerbates DSS-induced colitis and adenoma formation after AOM/DSS treatment. **(A)** Survival curves during the DSS treatment. Elevated mortality was observed in EPRAP-deficient (KO) mice, relative to WT (n = 18 each, *P < 0.01). **(B)** Percent changes in body weight (n = 16 [WT]; n = 17 [KO]). **(C)** Representative photographs of colons from each group. **(D)** Percent changes in colon length in WT and KO mice (n = 8 [WT]; n = 5 [KO]) in comparison to those of mice received DSS-free water. **(E)** Histological colitis scores (n = 10 each). **(F)** Representative H&E staining of rectal sections. **(G)** The numbers of F4/80-, Gr-1–, B220-, CD4-, and CD8-positive cells infiltrated in colonic tissues per high-power field (400× magnification) (n = 8 each). **(H)** The expression levels of TNF-α, IL-1β, IL-6, CXCL1, and MCP-1 protein in colonic tissue extracts from DSS-treated WT and KO mice (n = 15 [WT]; n = 9 [KO]). **(I)** Survival curves during the AOM/DSS treatment (n = 18 [WT]; n = 22 [KO], *P* < 0.05). **(J)** The number of colonic tumors per mouse, with size distribution (left) and total number (right) in AOM/DSS-treated WT and KO mice (n = 9 [WT]; 8 [KO]). **(K)** Representative photographs of colons at the end of AOM/DSS treatment. **(L)** Ki-67–positive cells in rectal polyps of AOM/DSS-treated WT and KO mice (left). KO mice exhibited greater numbers of Ki-67–positive cells than WT mice (right) (n = 5 each). **(M)** TUNEL assays on polyps of AOM/DSS-treated WT and KO mice. The arrows point to TUNEL-positive apoptotic cells: the insets show higher magnifications of selected regions (indicated by dashed boxes) (left). KO mice exhibited fewer TUNEL-positive cells than WT mice (right) (n = 5 each). All values represent means ± SEM. *P < 0.05, **P < 0.01 vs. WT mice. Scale bars: 100 μm.

The generation of mice lacking the gene encoding EPRAP enabled investigation of the role of EPRAP in colonic inflammation (S1 Fig). EPRAP-deficient mice were fertile and grew normally without apparent malformation. DSS-induced colitis is a widely used rodent model of human IBD [13]. After the administration of 2.5% DSS for 5 days and regular water for the following 16 days, EPRAP-deficient mice exhibited significantly higher mortality (Fig 1A) and markedly lower body weight (Fig 1B) relative to WT mice. In addition, EPRAP-deficient mice had reduced colon length (Fig 1C and 1D). Histopathologically, EPRAP-deficient mice treated with DSS exhibited more prominent crypt loss, as well as elevated infiltration by inflammatory cells: DSS-treated EPRAP-deficient mice had significantly higher histological damage scores [14] than did WT mice (Fig 1E and 1F). The colons of DSS-treated EPRAP-deficient mice showed significantly elevated accumulation of macrophages, neutrophils, B cells, CD4^+^ T cells, or CD8^+^ T cells (Figs 1G and S2A). Furthermore, the colons of DSS-treated EPRAP-deficient mice contained markedly higher levels of pro-inflammatory cytokines and chemokines such as TNF-α, IL-1β, IL-6, CXCL1, and MCP-1 compared to controls (Fig 1H). Markers for classically activated macrophages such as iNOS and CXCL10 also increased in EPRAP-deficient mice (S2B Fig). Thus, the colonic mucosa of EPRAP-deficient mice showed accentuated inflammatory damage caused by DSS treatment.

### EPRAP deficiency enhances polyp formation after AOM/DSS treatment

Colitis-associated tumorigenesis associates closely with the duration and severity of colonic inflammation [15]. Because EPRAP-deficient mice had more prominent colitis induced by DSS, we tested the hypothesis that EPRAP reduces colitis-associated tumorigenesis. WT and EPRAP-deficient mice were intraperitoneally injected with AOM, followed by three cycles of 5-day administration of 2% DSS. After this treatment, EPRAP-deficient mice exhibited a significantly higher mortality rate than WT mice (Fig 1I), with more prominent crypt loss and elevated histological damage scores (S3A and S3C Fig). The colons of AOM/DSS-treated EPRAP-deficient mice showed significantly increased accumulation of inflammatory cells including macrophages, neutrophils, B cells, CD4^+^ T cells, or CD8^+^ T cells (S3B and S3D Fig). Concomitantly, EPRAP-deficient mice developed a significantly greater number of colonic polyps at day 63 (Fig 1J and 1K). Furthermore, tumors of EPRAP-deficient mice had more Ki-67–positive (Fig 1L), but fewer TUNEL-positive cells, than those of WT mice (Fig 1M), indicating that EPRAP, either directly or indirectly, suppressed cell proliferation and promoted apoptosis during colitis-associated tumor development.

### EPRAP in bone marrow–derived cells attenuates colitis and colitis-induced tumorigenesis

**Fig 2:**
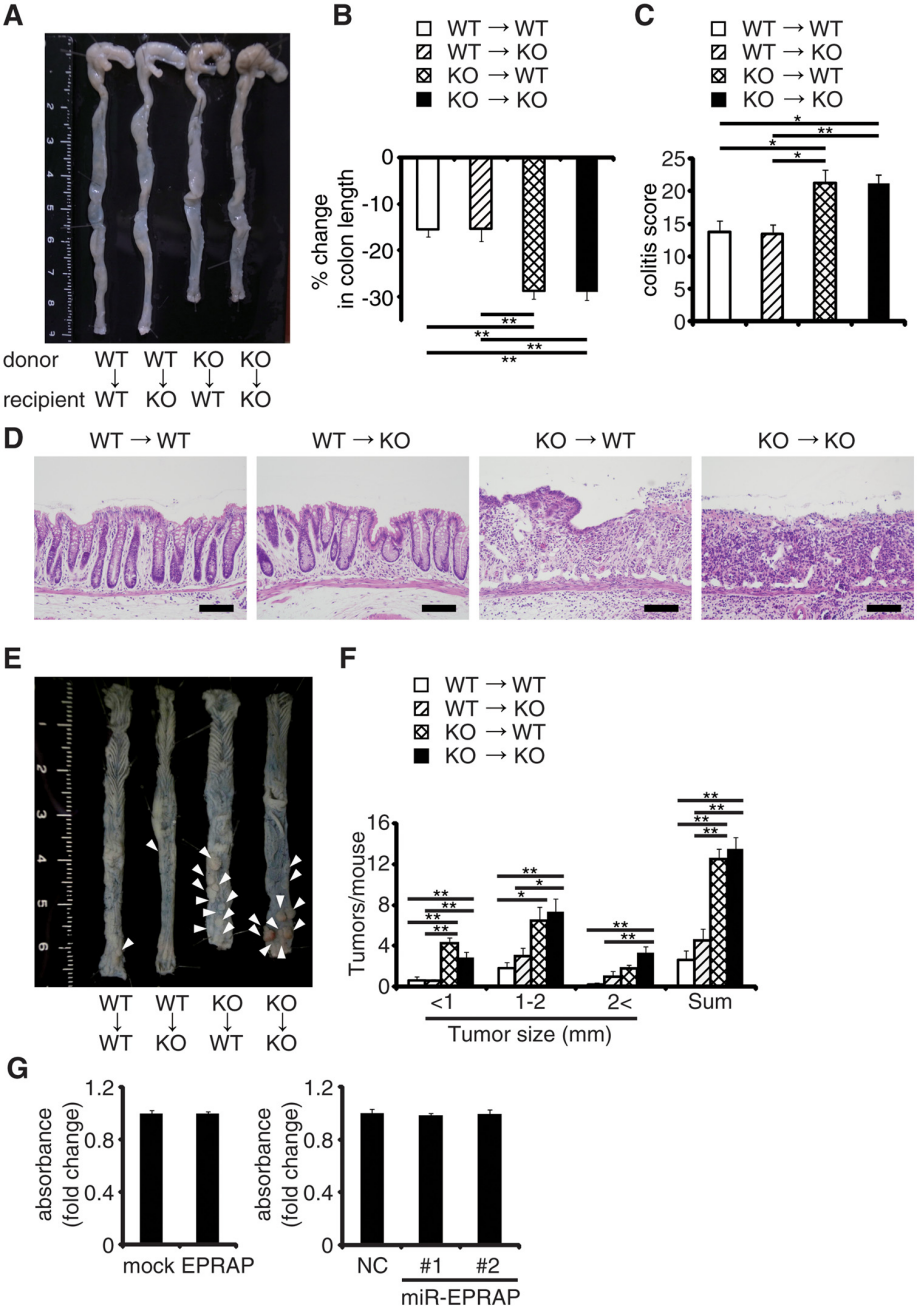
EPRAP in bone marrow–derived cells attenuates colitis and colitis-induced tumorigenesis. Four kinds of chimeric mice were generated by adoptive bone marrow transplantation: **(A)** Representative photographs of colons with adoptive bone marrow transplantation. **(B)** Percent changes in colon length expressed relative to DSS-free water controls in each group. (n = 4–8 each). **(C)** Histological colitis scores (n = 4–8 each). **(D)** Representative H&E staining of rectal sections. Scale bars: 100 μm. **(E)** Representative photographs of colons at the end of AOM/DSS treatment. **(F)** The number of colonic tumors per mouse, with the size distribution (left) and the total number (right) in each group (n = 4–9 each). **(G)** EPRAP did not directly affect cell proliferation. DLD-1 cell proliferation was determined by MTS assay (over expression of EPRAP, left; knockdown of EPRAP, right). Data represent fold induction of absorbance compared with mock or negative control. All values represent means ± SEM. *P < 0.05, **P < 0.01.

To determine which EPRAP-deficient cells contribute to the suppression of colitis and associated tumorigenesis, we used four kinds of chimeric mice generated by bone marrow transplantation. Following DSS treatment, WT and EPRAP-deficient recipients of EPRAP-deficient bone marrow exhibited greater colon shortening, crypt loss, content of inflammatory cells, and a higher histological damage score than WT or EPRAP-deficient recipients of WT bone marrow (Fig 2A–2D). Similarly, after AOM/DSS treatment, WT or EPRAP-deficient recipients of EPRAP-deficient bone marrow developed significantly more polyps (Fig 2E and 2F) than recipients of WT bone marrow. These observations indicated that EPRAP-expressing bone marrow cells predominated over epithelial cells in suppression of DSS-induced colitis and of AOM/DSS-induced tumorigenesis. In agreement with these *in vivo* findings in mice, human colon cancer cell lines with gain or loss of function of human EPRAP (S4 Fig) also showed that EPRAP expression did not affect cell proliferation (Fig 2G).

### Macrophages express EPRAP which suppresses their activation

Normal colonic tissues of WT mice showed negligible immunoreactive EPRAP; however, not epithelial cells but mononuclear cells in lamina propria and submucosal regions in the colonic tissues of DSS- or AOM/DSS-treated WT mice displayed abundant EPRAP (S5A and S5G Fig). Double-color immunofluorescence analyses revealed predominent EPRAP protein in F4/80-positive macrophages (S5B Fig), but not in CD4^+^ T cells, CD8^+^ T cells, dendritic cells, B cells, or NK cells (S5C Fig). Neutrophils also contained EPRAP (S5D Fig); however, EPRAP deficiency did not alter myeloperoxidase activity in the colonic tissues of DSS-treated mice (S5E Fig), suggesting that EPRAP deficiency in neutrophils does not play a major role in enhancing colitis and colitis-associated tumorigenesis. We further isolated lamina propria macrophages from DSS-treated mice by flow cytometry, and measured mRNAs that encode pro-inflammatory cytokines and chemokines by quantitative PCR. Lamina propria macrophages of EPRAP-deficient mice exhibited marked increases in the mRNAs corresponding to TNF-α, IL-1β, IL-6, CXCL1, and MCP-1 relative to those of WT mice (S5F Fig), indicating that EPRAP contributes critically to inhibiting macrophage activation and colonic inflammation.

### EPRAP deficiency impairs the anti-inflammatory effect of PGE_2_–EP4 signaling

**Fig 3:**
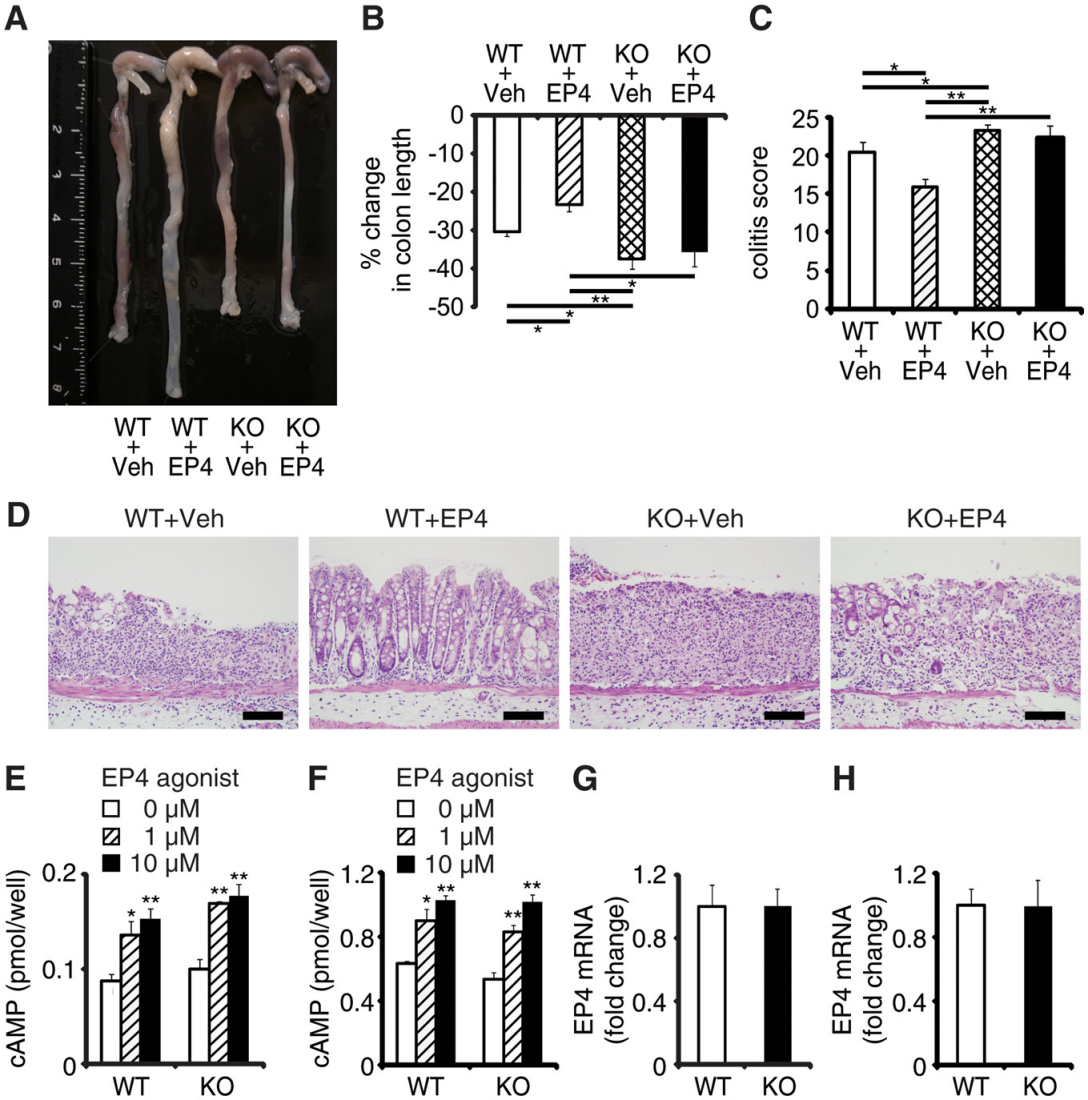
EPRAP deficiency impairs the anti-inflammatory effect of PGE/EP4. **(A)** Representative photographs of colons; “Veh” indicates vehicle and “EP4” indicates EP4 agonist. **(B)** Percent changes in colon length expressed relative to DSS-free water controls (n = 5–8 each; **P < 0.01 vs. WT + Veh). **(C)** Histological colitis scores (n = 5–8 each; *P < 0.05 vs. WT + Veh). **(D)** Representative H&E staining. Scale bars: 100 μm. **(E, F)** Peritoneal macrophages **(E)** and colonic epithelial cells **(F)** isolated from WT and KO mice were incubated with EP4 agonist. Intracellular cAMP levels were measured as described in Supplemental Methods (*P < 0.05 and **P < 0.01 vs. 0 μM). Figures are representative of five independent experiments performed in triplicate. **(G, H)** EP4 mRNA levels in peritoneal macrophages **(G)** and colonic epithelial cells **(H)** from WT and KO mice (n = 6 each). Data represent fold induction of mRNA expression compared with WT. All values represent means ± SEM.

EPRAP contributes to PGE_2_/EP4-mediated inhibition of inflammation of macrophages *in vitro* [12]. To determine whether EPRAP alters anti-inflammatory functions of EP4 signaling *in vivo*, we examined the pharmacological effects of ONO-AE1-329, a selective EP4 agonist, in DSS-induced colitis of WT and EPRAP-deficient mice. Administration of ONO-AE1-329 significantly decreased colon shortening, crypt loss, infiltration of inflammatory cells, and histological damage score in WT mice with DSS-induced colitis (Fig 3A–3D). In contrast, the EP4 agonist did not protect EPRAP-deficient mice from DSS-induced colitis, indicating that EP4 signaling improved colitis through EPRAP. Consistent with the results of previous *in vitro* experiments [12], administration of EP4 agonist decreased the phosphorylation of MEK and ERK in the stromal macrophages of DSS-treated WT but not EPRAP-deficient mice (S6 Fig). EP4 activation increases intracellular levels of cAMP, a major downstream effector of EP4 signaling. To test the involvement of EPRAP in the cAMP increase resulting from EP4 activation, we measured the levels of cAMP in peritoneal macrophages and colonic epithelial cells obtained from WT and EPRAP-deficient mice, with or without ONO-AE1-329 treatment. Cyclic AMP production induced by the EP4 agonist in both peritoneal macrophages (Fig 3E) and colonic epithelial cells (Fig 3F) did not differ significantly between WT and EPRAP-deficient mice. Furthermore, neither cell type showed a significant difference in the level of EP4 mRNA (Fig 3G and 3H). These data suggested that the anti-inflammatory function of EP4–EPRAP pathway does not involve cAMP increase.

### EPRAP inhibited p105 phosphorylation and MEK-ERK activation in stromal macrophages

**Fig 4:**
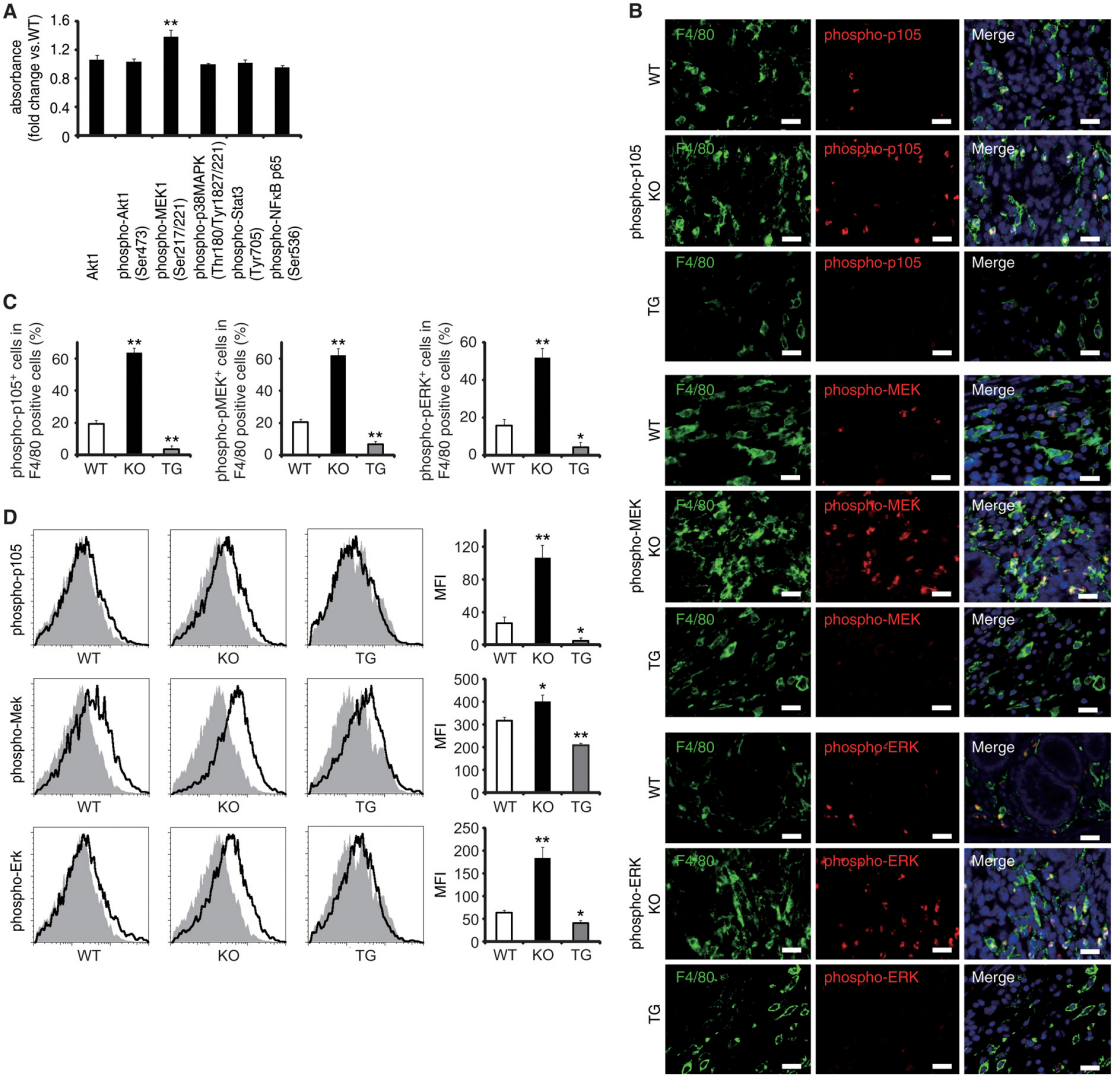
EPRAP inhibits p105 phosphorylation and MEK–ERK activation in stromal macrophages with DSS treatment. **(A)** The expression levels of Akt1, and phosphorylated forms of either Akt1, MEK1, p38MAPK, Stat3 or NF-κB p65 in colonic tissue extracts from DSS-treated KO mice compared to WT mice (n = 9 [WT]; n = 7 [KO]). **(B)** Double-color immunofluorescence analyses of rectal sections from DSS-treated WT, EPRAP-deficient (KO), and CD68–mEPRAP transgenic (TG) mice. Samples were immunostained with anti-F4/80 (green) and anti–phospho-p105 (red, top), anti–phospho-MEK (red, middle), or anti–phospho-ERK (red, bottom) antibodies. Scale bars: 20 μm. **(C)** The percentages of stained cells in F4/80 positive cells. (n = 5 each). **(D)** Lamina propria macrophages were isolated from the colonic tissues of WT, KO, and TG mice with DSS treatment. Cells were analyzed by flow cytometry, with detection of phospho-p105 (top), phospho-MEK (middle), or phospho-ERK (bottom) (open histograms). Cells were also stained with isotype control antibodies (solid histograms). Figures are representative histograms performed in quintuplicate (left). The levels of phosphorylated forms of p105 (top), MEK (middle), and ERK (bottom) in viable cells from WT, KO, and TG mice were determined as the geometric mean fluorescence intensity (MFI) of the target antibody minus the MFI of the isotype control (right) (n = 5 each). All values represent means ± SEM. **P < 0.01 vs. WT mice.

Via a direct interaction, EPRAP suppresses stimulus-induced phosphorylation and subsequent degradation of NF-κB1 p105, resulting in the inhibition of MEK and ERK activation in macrophages [12]. Study of WT and EPRAP-deficient mice affirmed that lacking EPRAP enhanced LPS-induced phosphorylation of p105, MEK and ERK (S7A and S7B Fig), with elevated mRNA production of TNF-α, IL-1β, IL-6, and CXCL1 (S7C Fig) in peritoneal macrophages *in vitro*. Pretreatment with the MEK inhibitor cancelled the increased expression of those pro-inflammatory molecules, indicating that EPRAP deficiency in macrophages causes unopposed MEK activation and subsequent inflammatory responses induced by LPS (S7D Fig). Indeed, phosphorylation levels of MEK increased significantly in inflamed colons isolated from EPRAP-deficient mice compared to those of WT mice following DSS treatment (Fig 4A). To corroborate EPRAP participation in this pathway in the context of DSS-induced colitis, we assessed the levels of the phosphorylated forms of p105, MEK, and ERK in stromal macrophages by immunohistochemistry and flow cytometry. Double-color immunofluorescence analyses demonstrated more prominent phosphorylated forms of p105, MEK, and ERK in the stromal macrophages of EPRAP-deficient mice than in those of WT mice (Fig 4B and 4C). Flow-cytometric analyses with lamina propria macrophages isolated from colitis lesions yielded similar results (Fig 4D): mean fluorescence intensity (MFI) indicated that the intracellular levels of the phosphorylated forms of each molecule varied inversely with the levels of EPRAP expression in macrophages. These observations suggested that EPRAP suppressed colitis through its interaction with NF-κB1 p105, thereby limiting the activation of the MEK–ERK MAPK pathway in stromal macrophages.

### EPRAP overexpression in macrophages suppresses colitis and colitis-associated tumorigenesis

**Fig 5:**
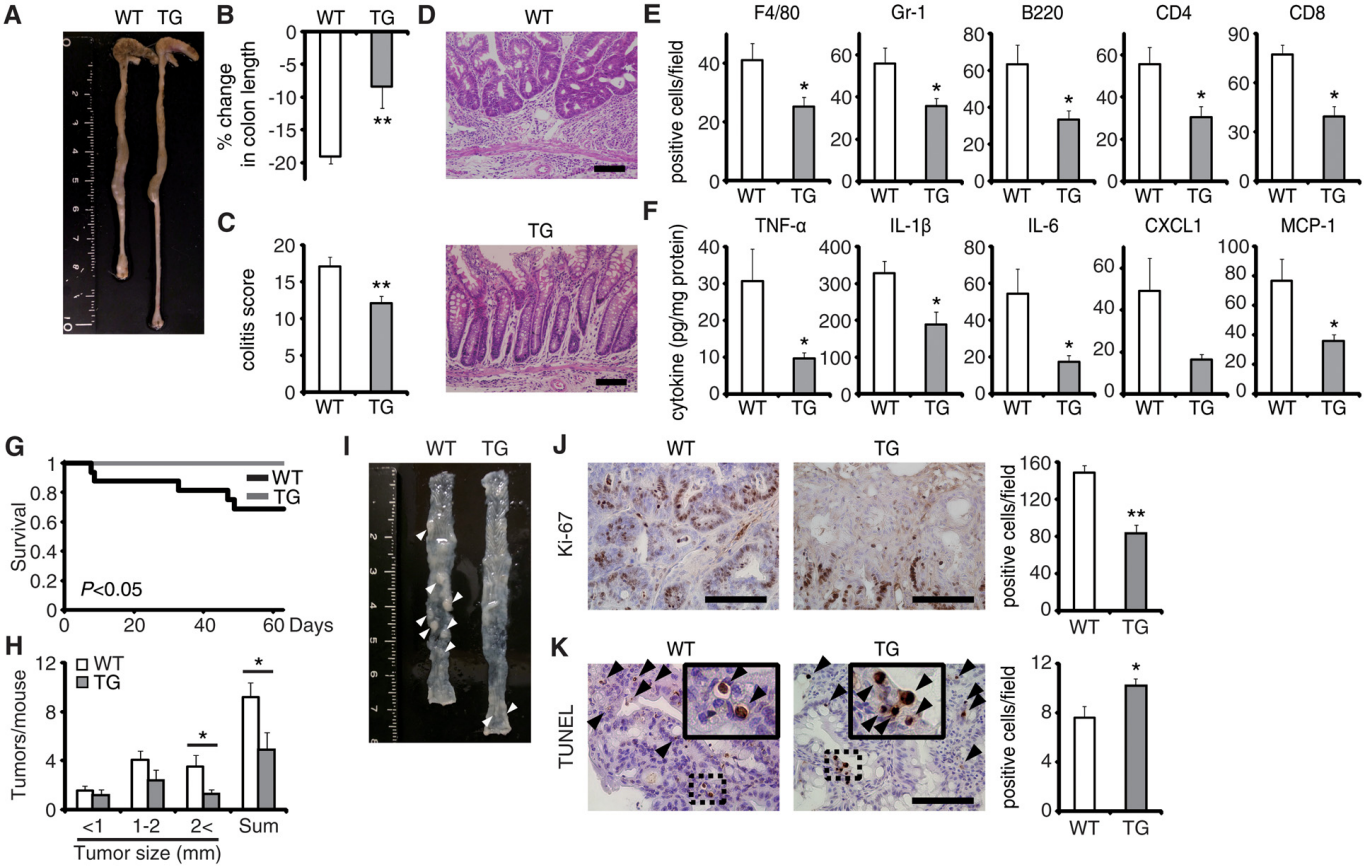
EPRAP overexpression in macrophages ameliorates DSS-induced colitis and colitis-associated tumorigenesis. **(A)** Representative photographs of colons at the end of DSS treatment; colons were obtained from WT and CD68–mEPRAP transgenic (TG) mice. **(B)** Percent changes in colon length expressed relative to DSS-free water controls (n = 8 [WT]; n = 6 [TG]). **(C)** Histological colitis scores (n = 10 [WT]; n = 8 [TG]). **(D)** Representative H&E staining of rectal sections. **(E)** The numbers of F4/80-, Gr-1–, B220-, CD4-, and CD8-positive cells infiltrated in colonic tissues of WT and TG mice, per high-power field (400× magnification) (n = 8 each). **(F)** The expression levels of TNF-α, IL-1β, IL-6, CXCL1, and MCP-1 protein in colonic tissue lysates from DSS-treated WT and TG mice (n = 15 [WT]; n = 8 [TG]). **(G)** Survival curves during the course of AOM/DSS treatment in WT and TG mice (n = 18 [WT]; n = 11 [TG]; *P* < 0.05). **(H)** The numbers of colonic polyps per mouse, with size distribution (left) and total number (right) in colonic tissues of AOM/DSS-treated WT and TG mice (n = 9 [WT]; n = 10 [TG]). **(I)** Representative photographs of colons at the end of AOM/DSS treatment. **(J)** Ki-67–positive cells in rectal polyps of AOM/DSS-treated WT and TG mice (left). TG mice exhibited markedly fewer Ki-67–positive cells than WT mice (n = 5 each) (right). **(K)** Representative photographs of TUNEL assay performed on rectal polyps of AOM/DSS-treated WT and TG mice. The arrows indicate TUNEL-positive apoptotic cells: the insets show higher magnifications of selected regions (indicated by dashed boxes) (left). TG mice exhibited more TUNEL-positive cells than WT mice (right) (n = 5 each). All values represent means ± SEM. *P < 0.05, **P < 0.01 vs. WT mice. Scale bars: 100 μm.

To verify the pivotal role of EPRAP in macrophages, we generated transgenic mice in which the murine CD68 promoter directed murine EPRAP expression (CD68–mEPRAP transgenic mice), leading to overexpression of EPRAP selectively in macrophages (S8 Fig). CD68–mEPRAP transgenic mice were fertile, accumulated body weight normally, and did not develop spontaneous diarrhea. During the course of DSS-induced colitis, WT and CD68–mEPRAP transgenic mice had no significant difference in mortality or body weight loss (S9A and S9B Fig); however, CD68–mEPRAP transgenic mice showed markedly reduced colon shortening (Fig 5A and 5B). Histological examination of the colon obtained from DSS-treated CD68–mEPRAP transgenic mice revealed reduced crypt loss and fewer inflammatory cells than in colons of WT mice (Fig 5C and 5D). Indeed, DSS-treated CD68–mEPRAP transgenic mice, had fewer neutrophils, B cells, CD4^+^ T cells, and CD8^+^ T cells as well as macrophages (Figs 5E and S9C), and reduced concentrations of TNF-α, IL-1β, IL-6, CXCL1, or MCP-1 (Fig 5F). As well, after 3 cycles of DSS treatment following intraperitoneal AOM injection, CD68–mEPRAP transgenic mice also exhibited less crypt loss, decreased infiltration of inflammatory cells with lower histological damage scores than WT mice (S10 Fig). In colitis-associated tumorigenesis, in contrast to WT mice, all CD68–mEPRAP transgenic mice survived the AOM/DSS treatment (Fig 5G), and CD68–mEPRAP transgenic mice developed fewer polyps (Fig 5H and 5I). In addition, polyps of CD68–mEPRAP transgenic mice contained significantly fewer Ki-67–positive cells (Fig 5J), but more TUNEL-positive cells, than those of WT mice (Fig 5K). These results indicated that forced expression of EPRAP in macrophages suppressed colitis and colitis-associated tumorigenesis.

Regarding the impact of EPRAP overexpression in macrophages on p105 phosphorylation and MEK–ERK activation, double-color immunofluorescence analyses demonstrated less phosphorylation of these molecules in stromal macrophages of CD68–mEPRAP transgenic mice with DSS treatment than in those of WT mice (Fig 4B and 4C). Flow-cytometric analyses with lamina propria macrophages isolated from colitis lesions yielded similar results (Fig 4D).

### EPRAP-positive macrophages were accumulated in the colon of ulcerative colitis patients

**Fig 6:**
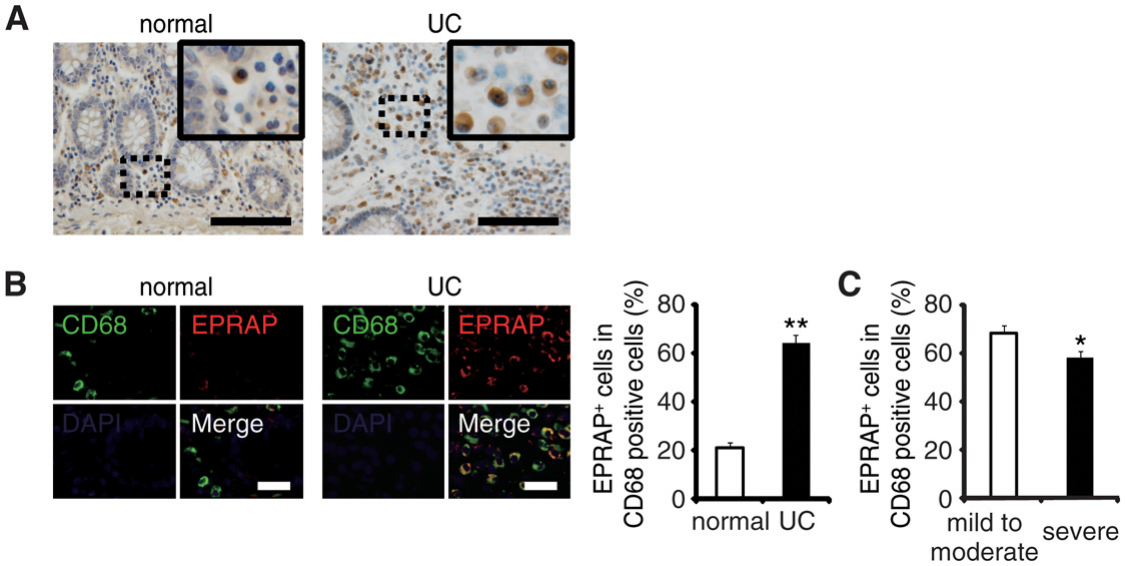
The colons of ulcerative colitis (UC) showed accumulation of EPRAP-positive macrophages. **(A)** Immunohistochemical staining for EPRAP in the sections of normal human and UC colons: the insets show higher magnifications of selected regions (indicated by dash boxes). Scale bars: 100 μm. **(B)** Double-color immunofluorescence analyses using sections of normal and UC colons were performed with anti-CD68 (green) and anti–EPRAP (red) antibodies (left). Scale bars: 20 μm. The percentage of EPRAP-positive cells in CD68-positive cells (right) (n = 20 [normal]; n = 20 [UC]). **P < 0.01 vs. normal colons. **(C)** Relationship between the percentage of EPRAP positive cells in CD68-positive macrophages and the clinical severity of UC patients (mild to moderate vs. severe) (n = 22 [mild to moderate]; n = 16 [severe]). *P < 0.05.

To examine the role of EPRAP on human IBD pathogenesis, we immunostained EPRAP in resected colonic specimens of ulcerative colitis (UC) patients. Normal colonic specimens obtained from another cohort showed few EPRAP-positive mononuclear cells; however, mononuclear cells in lamina propria in the colonic tissues of UC patients displayed a large number of EPRAP-positive cells (Fig 6A and 6B). Double-color immunofluorescence analyses suggested those EPRAP-positive cells are stromal macrophages (Fig 6B). Notably, there was an inverse relationship between the percentage of EPRAP-positive cells in stromal macrophages and the disease severity of the UC patients (Fig 6C), suggesting that inflammatory stimuli could induce EPRAP-positive macrophages to mitigate excess local immune responses; and less EPRAP expression in macrophages may cause more severe intestinal inflammation. Accordingly, phosphorylations or activations of p105, MEK and ERK were more prominent in EPRAP-negative mononuclear cells in lamina propria of human UC samples (S11 Fig).These data are consistent with DSS- or AOM/DSS-treated mouse colon, and suggest that EPRAP plays roles in human IBD.

## Discussion

Our previous studies showed that EPRAP, a novel EP4 receptor interactor, mediates the anti-inflammatory actions of EP4 in macrophages [9,12]. This study demonstrated that EPRAP expression is elevated in the stromal macrophages of both mouse and human colitis, and that EPRAP participates critically in suppressing colonic inflammation. Systemic or bone marrow–specific deficiency of EPRAP exacerbated DSS-induced colitis and increased AOM/DSS-induced polyp formation. In contrast, forced expression of EPRAP in macrophages or transplantation of EPRAP-sufficient bone marrow into EPRAP-deficient mice ameliorated these endpoints. Improvement of DSS-induced colitis by EP4 agonist administration required EPRAP, whereas cAMP, a downstream mediator of EP4, did not appear to contribute to the anti-inflammatory effect of EPRAP in the colon.

In mice treated with either DSS or AOM/DSS, EPRAP participated essentially in the regulation of pro-inflammatory gene expression and accumulation of inflammatory cells during mucosal inflammation and tumorigenesis. IBD associates with the development of colonic cancer [16], and EPRAP appeared to suppress colonic tumorigenesis indirectly by attenuating mucosal inflammation.

To determine which cell types are responsible for the suppression of colonic inflammation and tumorigenesis, we first examined cell proliferation assays using human colon cancer cell lines; however, EPRAP expression levels did not affect cell proliferation, suggesting that EPRAP does not play a role in epithelial or tumor cells. Consistent with this, EPRAP was not detectable by immunohistochemistry in colonic epithelial cells or lamina propria cells in the absence of inflammation; once these tissues were inflamed, however, lamina propria and submucosal inflammatory cells such as macrophages expressed EPRAP abundantly. Bone marrow transplantation experiments determined that EPRAP-expressing cells from this source mediated the reduction in bowel inflammation and a pivotal role for EPRAP-expressing cells in the stroma. Systemic or bone marrow-specific deficiency of EPRAP exacerbated colitis and colonic tumorigenesis; in contrast, forced expression of EPRAP in macrophages or restoration of EPRAP expression in bone marrow cells reversed these findings. Thus, EPRAP expression in macrophages mediates the suppression of inflammation and tumorigenesis in the colon.

In DSS-treated WT mice, neutrophils as well as macrophages in the colon expressed EPRAP. In human IBD, activated neutrophils accumulated within epithelial crypts produce reactive oxygen species, which induces oxidative stress and mucosal injury [17]; however, in this study, EPRAP deficiency did not affect myeloperoxidase activity, a marker for neutrophil oxidative stress. These data support the conclusion that EPRAP in macrophages, rather than in neutrophils, is critical for the suppression of colonic inflammation and tumorigenesis.

EP4 signaling attenuates colonic inflammation in mouse models of human IBD [4,5]. EP4 participates not only in reducing accumulation of macrophages but also in suppressing proliferation and activation of CD4^+^ T cells [5]. This study showed that the protective effect of an EP4 agonist against DSS-induced colitis depended largely on EPRAP. After DSS treatment, inflammatory cells as well as in colonic epithelial cells express EP4 [18]. Downstream of EP4, intracellular cAMP mediates many of the biological effects of EP4 signaling. For example, elevation of intracellular cAMP after EP receptor activation contributes to the defense of the gastrointestinal mucosa by stimulating mucin secretion from epithelial cells and regulating local immune responses [19,20]. Meanwhile, excessive secretion of mucin induced by the cAMP pathway promotes diarrhea. Furthermore, augmentation of intracellular cAMP by EP4 stimulation in vascular endothelial cells induces vasodilatation, evoking hypotension [7]. In this regard, the deletion of EPRAP affected neither the reduction in intracellular cAMP production mediated by EP4 activation nor EP4 expression. These results agree with our previous *in vitro* studies that showed that the anti-inflammatory effect of EP4–EPRAP in macrophages did not depend on intracellular cAMP [9,12]. Taken together, these data identify EPRAP signaling in macrophages as a target for IBD therapies that would avoid known unwanted actions of EP4 agonism in inflammatory diseases.

This study dissected the molecular mechanisms by which EPRAP suppresses colonic inflammation. Via a direct interaction, EPRAP in cultured macrophages inhibits stimulus-induced phosphorylation and subsequent degradation of NF-κB1 p105, thereby suppressing MAPK activation [12]. Indeed, NF-κB1 p105 is an important scaffold protein in MAPK signaling: p105 binds to tumor progression locus-2 (TPL2), a mitogen-activated protein kinase, and inhibits kinase activity [21]. In response to inflammatory stimulation, IκB kinase (IKK) complex phosphorylates p105, leading to proteolytic degradation of p105 and subsequent release of TPL2 [22]. TPL2 then directly phosphorylates and activates MEK, followed by phosphorylation and activation of ERK. ERK, a MAPK, phosphorylates a number of target proteins, and critically regulates pro-inflammatory activation in colitis and colitis-associated tumorigenesis [23]. Indeed, mice lacking the p105/p50 subunit have heightened susceptibility to colitis induced by *Helicobacter hepaticus* infection [24]. In addition, previous kinetic analyses using cell lysates from intestinal mucosa of IBD patients indicated marked acceleration of ATP-dependent degradation of p105 occurs in the presence of proteasomes from IBD patients [25]. This study demonstrated that EPRAP deficiency increased the proportions of the phosphorylated forms of p105, MEK, and ERK in stromal macrophages in DSS-induced colitis, whereas forced EPRAP expression had the opposite effect. In particular, EPRAP impaired the activation of MEK–ERK MAPK pathway, likely through p105, thereby decreasing the production of pro-inflammatory cytokines and chemokines in stromal macrophages and ultimately inhibiting colonic inflammation.

In summary, EPRAP in macrophages mediates attenuation of colonic inflammation by PGE_2_–EP4, and these functions do not depend on cAMP production. EPRAP in macrophages also suppressed tumorigenesis. These data identify EPRAP as a promising target for the treatment of IBD patients.

## Materials and Methods

### Ethics statement

All animal care and experiments were conducted following the guidelines for the Japan’s Act on Welfare and Management of Animals. The study protocol was approved by the Institutional Animal Care and Use Committees (IACUC)/ethics committee of Kyoto University. All surgery was performed when mice were anesthetized by 40 mg/kg of pentobarbital sodium (Kyoritsu Seiyaku, Tokyo, Japan), and all efforts were made to minimize suffering.

For immunohistochemistry of human samples, surgically resected specimens were obtained from ulcerative colitis (for colitis) or colorectal cancer patients (for surrounding normal colon) who had been admitted to Hyogo College of Medicine or Kyoto University Hospital, respectively. Written informed consents were obtained from all patients with the protocol approved by the Ethics Committee of Hyogo College of Medicine or Kyoto University Graduate School of Medicine in accordance with the ethical guidelines for epidemiological research by the Japanese Ministry of Education, Culture, Sports, Science and Technology and the Japanese Ministry of Health, Labour and Welfare as well as the principles expressed in the Declaration of Helsinki.

### Mice

Gene targeting in HK3i mouse ES cells derived from C57BL/6 embryos (Acc. No. CDB0852K: http://www.cdb.riken.jp/arg/protocol.html) generated EPRAP/Fem1a-deficient (KO) mice. The targeting vector was based on a modified pBluescript plasmid containing Mc1 DT-A-pA and a floxed Pr-NEO-pA cassette, and was designed to replace the mouse Eprap/Fem1a gene with a selection cassette (S1A Fig). PCR and Southern blot analysis identified homologous-recombinant ES clones. Chimeric mice were generated following aggregation of the targeted recombinant ES cells and transfer to recipient female mice. Chimeric males and C57BL/6 females were mated, and Southern blog analysis verified germline transmission and correct gene targeting. To produce EPRAP-deficient (KO) mice, we intercrossed heterozygous offspring. The primers used to identify WT and deleted alleles in PCR are listed in S1 Table. WT and KO littermates were housed individually until DSS or AOM/DSS treatment.

Mice overexpressing murine EPRAP/Fem1a under the control of the macrophage-specific promoter CD68 [CD68–mEPRAP transgenic (TG) mice] were generated as follows. The CD68–EPRAP/Fem1a transgene consists of the murine EPRAP/Fem1a cDNA downstream of the mouse CD68 promoter, which was derived from pDRIVE-mCD68 (InvivoGen, San Diego, CA, USA). We microinjected the transgene into fertilized C57BL/6 mouse eggs, and three lines were established from six founders. PCR, using a set of two primers (S1 Table), validated transgenic offspring. CD68-positive bone marrow cells were sorted by staining with FITC-labeled anti-CD68 antibody (BioLegend, San Diego, CA, USA), and quantitative PCR measured mouse EPRAP mRNA concentrations in each line. Mice with the highest level of EPRAP expression were bred to C57BL/6 mice to obtain heterozygous TG mice and WT littermates and used for the experiments described in this article.

### Colitis and colitis-associated tumorigenesis

For DSS-induced colitis, 8–10-week-old male WT, KO, and TG mice were given 2.5% (w/v) DSS (mol wt, 36,000–50,000; MP Biomedicals, Irvine, CA, USA) in their drinking water for 5 days, followed by a recovery period with regular drinking water through the end of the experiment (day 21). Control mice received DSS-free drinking water. For AOM/DSS-induced inflammatory tumorigenesis, 8–10-week-old male WT, KO, and TG mice received intraperitoneal injection of 12 mg/kg AOM (Sigma-Aldrich, St. Louis, MO, USA) before three cycles of DSS administration as described above. All mice were maintained on food and water ad libitum, and were age-matched as well as co-housed for all experiments.

### Administration of EP4 agonist

Eight- to ten-week-old male WT and EPRAP-deficient (KO) mice were each divided into two groups, EP4 agonist–treated and vehicle-treated. All four groups of mice (EP4 agonist–treated WT, vehicle-treated WT, EP4 agonist–treated KO, and vehicle-treated KO) received 2.5% (w/v) DSS in their drinking water *ad libitum* for 7 days [5]. Mice consumed either 100 μg/kg of ONO-AE1-329 (Ono Pharmaceutical Co. Ltd., Osaka, Japan) or vehicle daily via the transanal route for 8 consecutive days, starting the day before DSS administration.

### Generation of bone marrow chimeric mice

Four- to eight-week-old male C57BL/6 wild type (WT) or EPRAP-deficient (KO) recipient mice were irradiated with X-rays (10 Gy) and injected intravenously with 6–14×10^6^ bone marrow cells derived from femurs and tibias of adult WT or KO mice. Quantitative PCR verified peripheral blood chimerism: circulating blood cells were isolated and EPRAP mRNA expression was determined (see RNA isolation and quantitative PCR).

### Antibodies and histopathological and immunohistochemical analyses

Rabbit polyclonal antibody against mouse EPRAP was raised by immunization with keyhole limpet hemocyanin–conjugated synthetic peptides corresponding to amino-acid residues 244–260, 330–346, and 629–645 of murine EPRAP. For immunohistochemistry of mouse samples, paraffin-embedded sections were routinely stained with one of the following antibodies: rat anti-F4/80 (Abcam, Cambridge, MA, USA), rat anti–Gr-1 (eBioscience, San Diego, CA, USA), rat anti-B220 (eBioscience), rat anti-CD4 (eBioscience), rat anti-CD8 (Abcam), hamster anti-CD11c (eBioscience), mouse anti-NCAM (Abcam), rabbit anti–phospho-NF-κB p105 (Ser933) (Cell Signaling, Boston, MA, USA), rabbit anti–phospho-MEK1/2 (Ser221) (Cell Signaling), or rabbit anti–phospho-p44/42 MAPK (Thr202/Tyr204) (Cell Signaling), or rabbit anti-EPRAP.

For immunohistochemistry of human samples, paraffin-embedded sections were stained with rat anti-CD68 (Abcam), goat anti-FEM1A (Abcam), and rabbit anti–phospho-NF-κB p105 (Ser933), rabbit anti–phospho-MEK1/2 (Ser221), or rabbit anti–phospho-p44/42 MAPK (Thr202/Tyr204) antibodies. Staining with non-immune rat or rabbit IgG served as a negative control for each experiment.

### Histological assessment of mouse colitis

A validated scoring scheme assessed colitis. In brief, histological scoring was based on three parameters. Inflammation severity was scored as follows: 0, none; 1, mild; 2, moderate; 3, severe. Inflammation extent was scored as follows: 0, none; 1, mucosa; 2, mucosa and submucosa; 3, transmural. Crypt damage was scored as follows: 0, none; 1, basal 1/3 damaged; 2, basal 2/3 damaged; 3, crypts lost, surface epithelium present; 4, crypts and surface epithelium lost. Inflammation severity score, inflammation extent score, and crypt damage score were each multiplied by percent involvement (0, 0%; 1, 1–25%; 2, 26–50%; 3, 51–75%; 4, 76–100%), and the resultant products were summed to yield a histological score ranging from 0 to 40.

### Clinical parameters of ulcerative colitis patients

Disease severity was determined based on the guidelines for the management of ulcerative colitis in Japan (modification of Truelove and Witts’ criteria) proposed by the Research Committee of Inflammatory Bowel Disease [26]. The severity criteria is listed in S1 Table.

### TUNEL assay

To detect apoptotic cells, paraffin-embedded sections were stained using the ApopTag in situ apoptosis detection kit (Millipore, Billerica, MA, USA). Five different areas per tissue section were analyzed using BZ-H2C (Keyence, Osaka, Japan).

### Quantitative determination of cAMP

Peritoneal macrophages or colonic epithelial cells were harvested from 8-week-old WT, KO, and CD68–mEPRAP transgenic (TG) mice, and 3×10^5^ cells were seeded into individual wells of a 96-well dish. After 10-minute treatment with different concentration of ONO-AE1-329 or vehicle, intracellular cAMP levels were determined using the Cyclic AMP EIA Kit (Cayman Chemical).

### Cytometric bead assay and ELISA

Colonic tissues were removed and homogenized in 1% NP-40 supplemented with protease inhibitor cocktail and PhosSTOP (Roche Diagnostics, Indianapolis, IN, USA). The BD CBA assay (BD Bioscience, San Jose, CA, USA) measured quantitative determinations of TNF-α, IL-6, and MCP-1 concentrations in the supernatants of tissue homogenates. Quantikine ELISA Immunoassay (R&D Systems, Minneapolis, MN, USA) measured IL-1β and CXCL1 concentrations.

### Myeloperoxidase activity

The Myeloperoxidase (MPO) Activity Colorimetric Assay Kit (BioVision, Inc., Milpitas, CA, USA) measured MPO activity. MPO activity was compensated by the protein concentration of lysates.

### Phosphorylation assay (colonic tissues)

Lysates of colonic tissue extracts from DSS-treated WT and KO mice were assayed at a protein of 0.5 mg/ml or 0.05 mg/ml with the PathScan Signaling Nodes Multi-Target Sandwich ELISA Kit (Cell Signaling) according to the manufacture’s protocol.

### Phosphorylation assay (peritoneal macrophages)

Peritoneal macrophages were harvested from 10–12-week-old WT and KO mice and 1.5×10^6^ cells were seeded into individual wells of 48-well dishes. After 1 hour of treatment with 1 μg/ml of lipopolysaccharides (E. Coli 055:B5; LPS) (Calbiochem, San Diego, CA, USA) or vehicle, endogenous levels of phospho-MEK and phospho-ERK were determined at a protein of 0.3 mg/ml using the PathScan Phospho-MEK1 (Ser217/221) Chemiluminescent Sandwich ELISA Kit and the PathScan Phospho-p44/42 MAPK (Thr202/Tyr204) Sandwich ELISA Kit (Cell Signaling) according to the manufacture’s protocol. Immunoblot analyses were also performed using the whole cell lysates of WT and KO peritoneal macrophages. Antibodies against phospho-NF-κB p105 (Ser933), phospho-MEK1/2 (Ser217/221), MEK1/2, phospho-p44/42 MAPK (Thr202/Tyr204), p44/42 MAPK (Erk1/2) were from Cell Signaling Technology: anti-mouse p105/p50 antibody was from Abcom. For quantitative PCR, peritoneal macrophages were treated with or without LPS, followed by total RNA isolation as described in the Materials and Methods. To test the effects of pharmacologic inhibition of MEK, cells were pretreated with U0126 (Cell Signaling) at 10 μM for 1 hour prior to LPS treatment.

### Isolation of lamina propria mononuclear cells and colonic epithelial cells, and flow-cytometry analysis

Lamina propria mononuclear cells and colonic epithelial cells were isolated as previously described [27]. Briefly, colonic tissues were incubated in HBSS containing 5 mM EDTA and 1 mM DTT at 37°C with shaking, and then filtered through a 100-μm cell strainer (BD Biosciences). Colonic epithelial cells were harvested from the sediment of the flow-through at this step. To isolate lamina propria mononuclear cells, the remaining pre-digested colonic tissues were minced and incubated with PBS containing 500 μg/ml of collagenase D (Roche), 500 μg/ml of DNase I (Sigma-Aldrich), and 3 mg/ml of dispase II (Eidia, Ibaraki, Japan) at 37°C with shaking. After removing all remaining cell clumps by passing the suspension through a 40-μm cell strainer (BD Biosciences), cells were resuspended with 3% (v/v) FCS in PBS. CD45^+^CD11b^+^ macrophages were isolated using a FACSAria II flow cytometer (BD Biosciences), following staining of cell suspensions with FITC-labeled anti-CD45 (eBioscience) and PerCP–Cy5.5-labeled anti-CD11b (eBioscience). For analyses of the levels of phosphorylated forms of p105, MEK and ERK, freshly isolated macrophages were fixed in 4% paraformaldehyde/PBS, permeabilized in 0.5% Triton X-100/PBS, and stained with rabbit anti–phospho-p105, anti–phospho-MEK1/2, or anti–phospho-p44/42 MAPK antibody, followed by incubation with PE-conjugated donkey anti-rabbit IgG (eBioscience). Flow cytometry was performed using FACSAria II and analyzed using the FlowJo software (Tree Star Inc., Ashland, OR, USA).

### RNA isolation and quantitative PCR

Trizol Reagent (Invitrogen) was used to extract total RNA, Superscript III (Invitrogen) was used to synthesize single-stranded cDNA. The mRNA level for each target gene was determined by SYBR Green–based quantitative PCR using a LightCycler 480 system (Roche). Primer sequences are shown in S2 Table. Data were normalized using GAPDH as a reference gene. All reactions were performed in triplicate.

### Knockdown of EPRAP

To knockdown endogenous EPRAP expression in colon cancer cells (DLD-1, ATCC No.: CCL-221), we used the BLOCK–iTPol II miR RNAi Expression Vector Kit with EmGFP (Invitrogen). miRNA-expressing plasmids were constructed according to the manufacturer’s protocol. Target sequences were as follows: miR–EPRAP #1, 5′-ACCAACCGAAAGCTATGCAAG-3′; miR–EPRAP #2, 5′-AAACCAACCGAAAGCTATGCA-3′. We used the pcDNA6.2/EmGFP–miR-negative control vector (miR–NC from Invitrogen) as a negative control. Plasmid transfection experiments were performed using Lipofectamine 2000 reagent (Invitrogen).

### In vitro cell proliferation analysis

Following over expression [12] or knockdown of EPRAP, cell proliferation was measured with the CellTiter 96 AQueous One Solution Cell Proliferation Assay (MTS; Promega, Madison, WI). Briefly, 5 × 10^3^ cells were seeded in 96–well plates and cultured in the growth medium containing 10% fetal bovine serum for 0 h, 48 h, or 72 h. Then, absorbance at 492 nm was measured according to the manufacture’s protocol. All experiments were performed in octuplicate, and the results are shown as mean ± SEM of values.

### Statistics

Data are presented as means ± SEM. Statistical comparisons between groups were made using Student’s *t* test or one-way ANOVA followed by Tukey-Kramer analysis. *P* values less than 0.05 were considered statistically significant.

## Supporting Information

S1 Fig Generation of EPRAP-deficient mice. **(A)** Targeting strategy for the generation of the EPRAP-deficient strain. **(B)** Genotype mapping of WT (+/+), heterozygous (+/-), and homozygous mutant (-/-) mice by genomic Southern blot analysis. Digestion of genomic DNA with AseI produced an 18-kb fragment from the WT allele, and a 10-kb fragment from the mutant allele, of the Eprap/Fem1a gene. **(C)** The EPRAP mRNA levels of WT (+/+), heterozygous (+/-), and homozygous (-/-) mutant mice in normal colonic extracts were analyzed by quantitative PCR (n = 3–4 each). Data represent fold induction of mRNA expression compared with WT (+/+). (D) A specific antibody against murine EPRAP was generated. EPRAP was immunostained with this antibody, using rectal sections obtained from DSS-treated WT (+/+) and homozygous EPRAP-deficient (-/-) mice.
(TIF)

S2 Fig EPRAP deficiency exacerbates DSS-induced colitis. **(A**) Immunohistochemical staining to detect F4/80, Gr-1, B220, CD4, and CD8 in rectal sections of DSS-treated WT and EPRAP-deficient (KO) mice. (**B**) The mRNA levels of iNOS and CXCL10 in colonic stromal macrophages of DSS-treated WT and KO mice (n = 7 [WT]; n = 5 [KO]). Data represent fold induction of mRNA expression compared with WT.
(TIF)

S3 Fig EPRAP deficiency exacerbates AOM/DSS colitis. **(A)** H & E staining in rectal sections (non-polyp lesion) of AOM/DSS-treated WT and KO mice. **(B)** Immunohistochemical staining to detect F4/80, Gr-1, B220, CD4, and CD8 in rectal sections (non-polyp lesion) of AOM/DSS-treated WT and KO mice. **(C)** Histological colitis score in rectal sections (non-polyp lesion) of AOM/DSS-treated WT and KO mice (n = 9 [WT]; n = 5 [KO]). **(D)** The numbers of F4/80-, Gr-1-, B220-, CD4-, and CD8-positive cells infiltrated in colonic tissues per high-power field (400× magnification) in rectal sections (non-polyp lesion) of AOM/DSS-treated WT and KO mice (n = 9 [WT]; n = 5 [KO]). All values represent means ± SEM. *P < 0.05, **P < 0.01 vs. WT mice. Scale bars: 100 μm.
(TIF)

S4 Fig EPRAP overexpression and knockdown. **(A, B)** DLD-1 cells were transiently transfected with V5-tagged human EPRAP expression construct or mock plasmid. Immunoblot analysis showing the enforced expression of recombinant EPRAP **(A)**: quantitative real-time PCR showing increased levels of total cellular EPRAP mRNA (n = 4 each). **P < 0.01 vs. mock **(B)**. **(C)** EPRAP gene knockdown experiments were performed as described in the Materials and Methods. Testing the efficiency of the gene silencing entailed quantitative real time PCR analysis (n = 4 each). *P < 0.05 vs. negative control.
(TIF)

S5 Fig EPRAP is present in, and deactivates, macrophages in lamina propria and submucosa. **(A)** Immunohistochemical detection of EPRAP-expressing cells. The colonic tissues were obtained from WT mice subjected to no treatment (drug-free water; normal colon), DSS treatment, or AOM/DSS treatment. Scale bars: 100 μm. **(B)** Double–color immunofluorescence staining of rectal sections from control-, DSS-, or AOM/DSS-treated WT mice was performed with a combination of anti-EPRAP (red) and anti-F4/80 (green) antibodies. Scale bars: 20 μm. **(C)** Double–color immunofluorescence staining of rectal sections from DSS-treated WT mice was performed using a combination of anti-EPRAP (red) and anti-CD4, anti-CD8, anti-CD11c, anti-B220, or anti-NCAM (green) antibodies. Scale bars: 20 μm. **(D)** Double–color immunofluorescence staining of rectal sections from DSS-treated WT mice was performed using a combination of anti-EPRAP (red) and anti–Gr-1 (green) antibodies. Scale bars: 20 μm. **(E)** MPO activity was measured in protein extracts from colonic tissues from DSS-treated WT and EPRAP-deficient (KO) mice (n = 6 [WT]; n = 4 [KO]). MPO activity (left) and MPO activity compensated by the numbers of Gr-1–positive cells infiltrated in colonic tissues per high-power field (Fig 1G) (right). **(F)** The mRNA levels of TNF-α, IL-1β, CXCL1, and MCP-1 in colonic stromal macrophages of DSS-treated WT and EPRAP-deficient (KO) mice (n = 7 [WT]; n = 5 [KO]). **(G)** The EPRAP mRNA levels of lamina propria macrophages (LPMC) and epithelial cells isolated from the colonic tissues of WT mice with DSS treatment. Data represent fold induction of mRNA expression compared with WT. *P < 0.05, **P < 0.01 vs. WT mice.
(TIF)

S6 Fig EP4 signaling ameliorates MEK and ERK activation through EPRAP in sromal macrophages with DSS treatment. **(A)** Double-color immunofluorescence staining of rectal sections from DSS-treated WT and EPRAP deficient mice with or without EP4 agonist treatment (as described in the Fig 3 legend) was performed with anti-F4/80 (green) and anti–phospho-MEK (red) (left), anti–phospho-ERK (red) (right). Scale bars: 20 μm. **(B)** The percentages of phospho-MEK (left) or phospho-ERK (right) positive cells in F4/80 positive macrophages (n = 5 each).
(TIF)

S7 Fig The lack of EPRAP enhances lipopolysaccharide (LPS)-induced phosphorylation of p105, MEK and ERK in peritoneal macrophages. Peritoneal macrophages were isolated from 10–12-week-old of WT or KO mice. Cells were incubated with LPS or vehicle for one hour, followed by whole cell extraction. **(A)** Immunoblot analyses were performed to examine the levels of phosphorylated forms of p105, MEK and ERK. Figures are representative of three independent experiments. **(B)** The expression levels of phosphorylated forms of MEK and ERK were examined (n = 4 each). **(C)** The mRNA levels of TNF-α, IL-1β, IL-6, and CXCL1 in peritoneal macrophages of WT or KO mice (n = 7–9 each). **(D)** The mRNA levels of TNF-α, IL-1β, IL-6, and CXCL1 in peritoneal macrophages of WT or KO mice pre-treated with MEK inhibitor (U0126) (n = 6 each). Data represent fold induction of mRNA expression of LPS-treated cells compared with that of vehicle-treated cells. *P < 0.05, **P < 0.01 vs. WT mice.
(TIF)

S8 Fig CD68-mEPRAP transgenic mice. **(A)** Double–color immunofluorescence staining of rectal sections from WT and CD68–mEPRAP transgenic (TG) mice was performed with a combination of anti-EPRAP (red) and anti-F4/80 (green) antibodies. **(B)** The mRNA levels of EPRAP in CD68-positive bone marrow cells (n = 4 each). Data represent fold induction of mRNA expression compared with WT. **P < 0.01 vs. WT mice.
(TIF)

S9 Fig EPRAP overexpression in macrophages ameliorated cellular infiltration in DSS-induced colitis. **(A)** Survival curves during the DSS treatment. WT and TG mice had no significant difference in mortality (n = 18 [WT]; n = 9 [TG]). **(B)** Percent changes in body weight (n = 16 [WT]; n = 8 [TG]). WT and TG mice had no significant difference. **(C)** Immunohistochemical staining to detect F4/80, Gr-1, B220, CD4, and CD8 in rectal sections of DSS-treated WT and TG mice.
(TIF)

S10 Fig EPRAP overexpression in macrophages ameliorated AOM-DSS-induced colitis. **(A)** H & E staining in rectal sections (non-polyp lesion) of AOM/DSS-treated WT and TG mice. **(B)** Immunohistochemical staining to detect F4/80, Gr-1, B220, CD4, and CD8 in rectal sections (non-polyp lesion) of AOM/DSS-treated WT and TG mice. **(C)** Histological colitis score in rectal sections (non-polyp lesion) of AOM/DSS-treated WT and KO mice (n = 9 [WT]; n = 8 [TG]). **(D)** The numbers of F4/80-, Gr-1–, B220-, CD4-, and CD8-positive cells infiltrated in colonic tissues per high-power field (400× magnification) in rectal sections (non-polyp lesion) of AOM/DSS-treated WT and TG mice (n = 9 [WT]; n = 8 [TG]). All values represent means ± SEM. **P < 0.01 vs. WT mice. Scale bars: 100 μm.
(TIF)

S11 Fig p105 phosphorylation and MEK-ERK activation in EPRAP-positive cells in the colons of human ulcerative colitis (UC) patients. **(A)** Double–color immunofluorescence staining of colonic sections of UC patients was performed with a combination of anti-EPRAP (green) and anti–phospho-p105 (red, top), anti–phospho-MEK (red, middle), or anti–phospho-ERK (red, bottom) antibodies. Scale bars: 20 μm.
(TIF)

S1 Table The criterion for severity of ulcerative colitis. (DOCX)

S2 Table Primers used in this study. (DOCX)
