# Supplementary figures and images for: EP4 Receptor–Associated Protein in Macrophages Ameliorates Colitis and Colitis-Associated Tumorigenesis
Source: PLoS Genet. 2015 Oct 6;11(10):e1005542. doi: 10.1371/journal.pgen.1005542 (PMC4595503; doi:10.1371/journal.pgen.1005542)

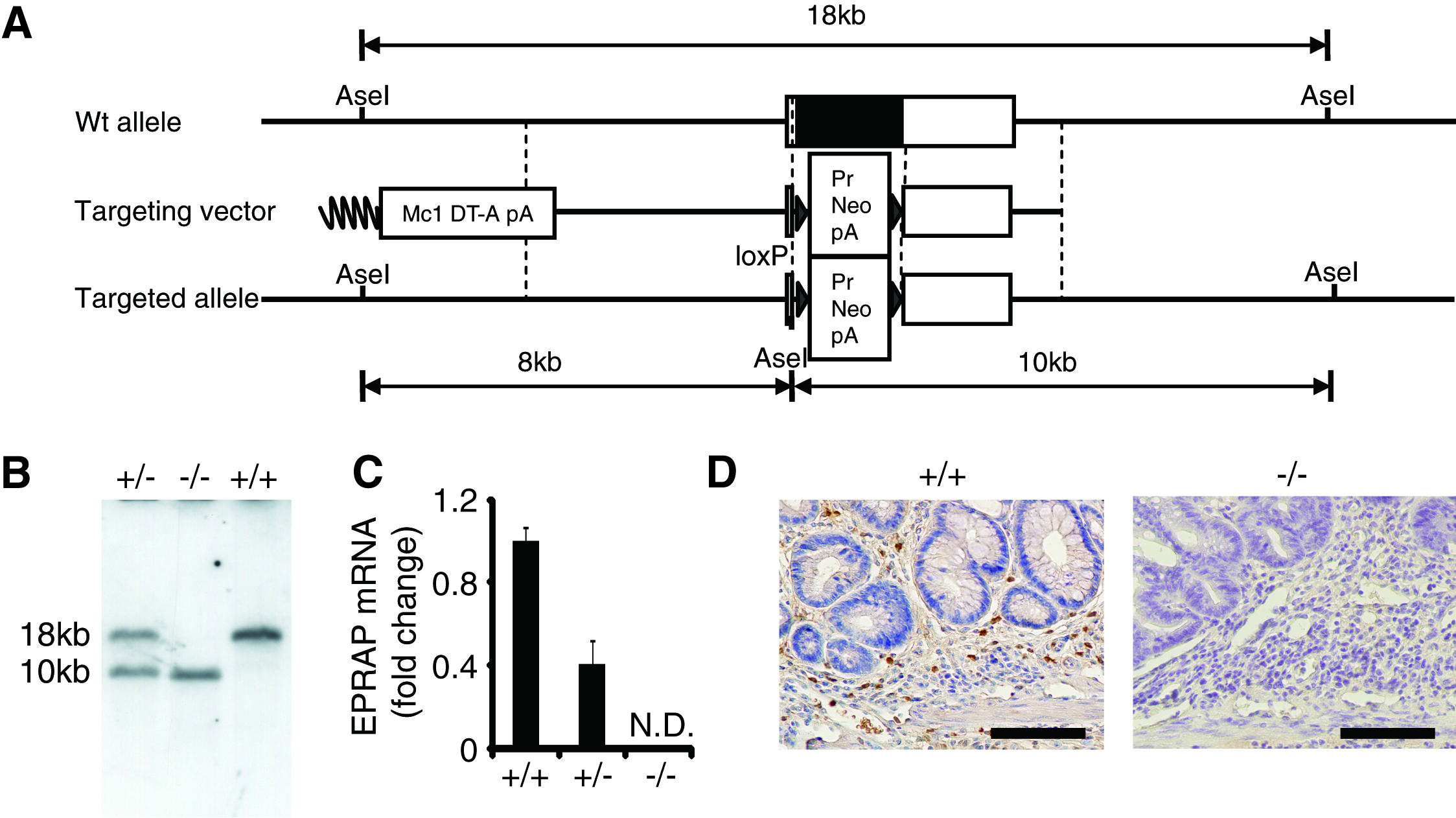

Supplement: S1 Fig — (A) Targeting strategy for the generation of the EPRAP-deficient strain. (B) Genotype mapping of WT (+/+), heterozygous (+/-), and homozygous mutant (-/-) mice by genomic Southern blot analysis. Digestion of genomic DNA with AseI produced an 18-kb fragment from the WT allele, and a 10-kb fragment from the mutant allele, of the Eprap/Fem1a gene. (C) The EPRAP mRNA levels of WT (+/+), heterozygous (+/-), and homozygous (-/-) mutant mice in normal colonic extracts were analyzed by quantitative PCR (n = 3–4 each). Data represent fold induction of mRNA expression compared with WT (+/+). (D) A specific antibody against murine EPRAP was generated. EPRAP was immunostained with this antibody, using rectal sections obtained from DSS-treated WT (+/+) and homozygous EPRAP-deficient (-/-) mice. (TIF) [file pgen.1005542.s001.tif]

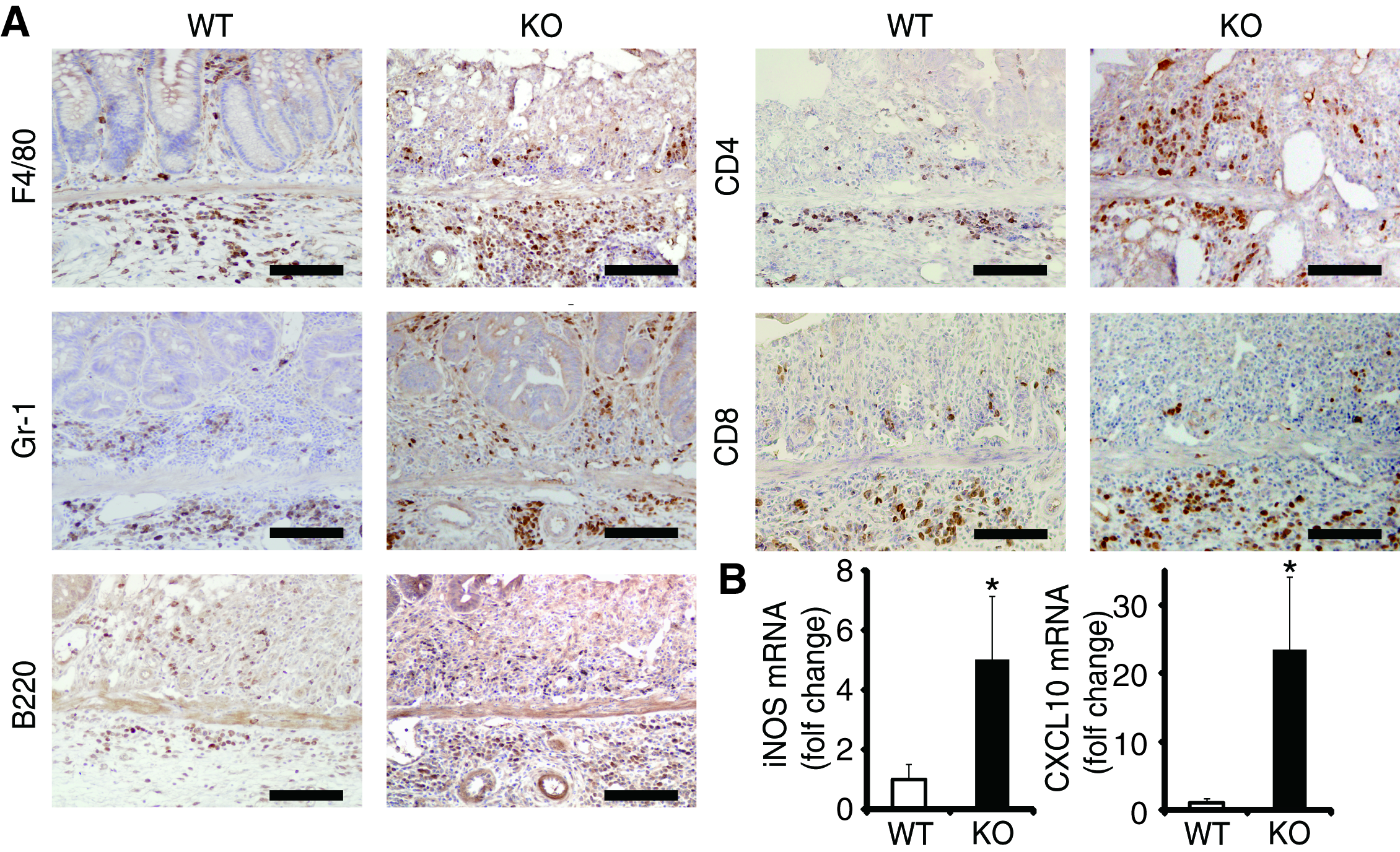

Supplement: S2 Fig — (A) Immunohistochemical staining to detect F4/80, Gr-1, B220, CD4, and CD8 in rectal sections of DSS-treated WT and EPRAP-deficient (KO) mice. (B) The mRNA levels of iNOS and CXCL10 in colonic stromal macrophages of DSS-treated WT and KO mice (n = 7 [WT]; n = 5 [KO]). Data represent fold induction of mRNA expression compared with WT. (TIF) [file pgen.1005542.s002.tif]

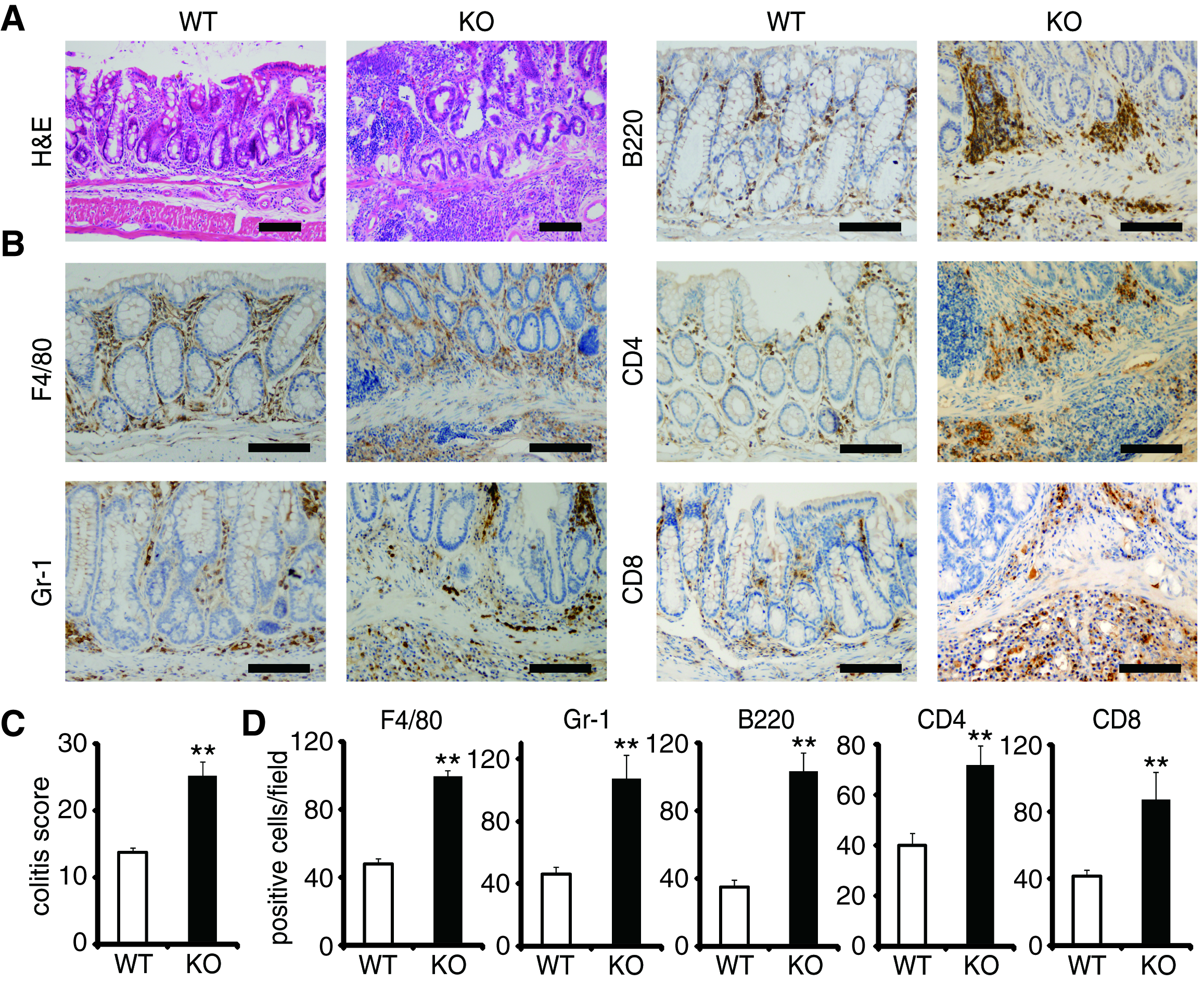

Supplement: S3 Fig — (A) H & E staining in rectal sections (non-polyp lesion) of AOM/DSS-treated WT and KO mice. (B) Immunohistochemical staining to detect F4/80, Gr-1, B220, CD4, and CD8 in rectal sections (non-polyp lesion) of AOM/DSS-treated WT and KO mice. (C) Histological colitis score in rectal sections (non-polyp lesion) of AOM/DSS-treated WT and KO mice (n = 9 [WT]; n = 5 [KO]). (D) The numbers of F4/80-, Gr-1-, B220-, CD4-, and CD8-positive cells infiltrated in colonic tissues per high-power field (400× magnification) in rectal sections (non-polyp lesion) of AOM/DSS-treated WT and KO mice (n = 9 [WT]; n = 5 [KO]). All values represent means ± SEM. *P < 0.05, **P < 0.01 vs. WT mice. Scale bars: 100 μm. (TIF) [file pgen.1005542.s003.tif]

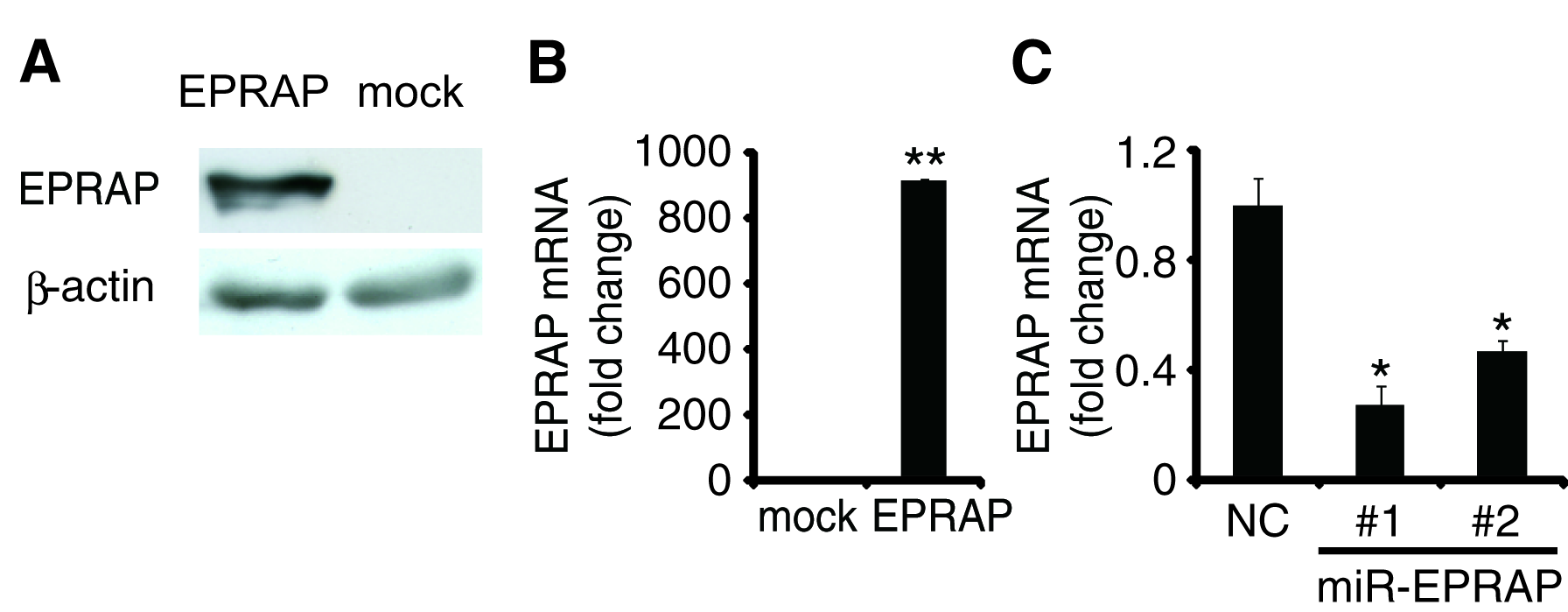

Supplement: S4 Fig — (A, B) DLD-1 cells were transiently transfected with V5-tagged human EPRAP expression construct or mock plasmid. Immunoblot analysis showing the enforced expression of recombinant EPRAP (A): quantitative real-time PCR showing increased levels of total cellular EPRAP mRNA (n = 4 each). **P < 0.01 vs. mock (B). (C) EPRAP gene knockdown experiments were performed as described in the Materials and Methods. Testing the efficiency of the gene silencing entailed quantitative real time PCR analysis (n = 4 each). *P < 0.05 vs. negative control. (TIF) [file pgen.1005542.s004.tif]

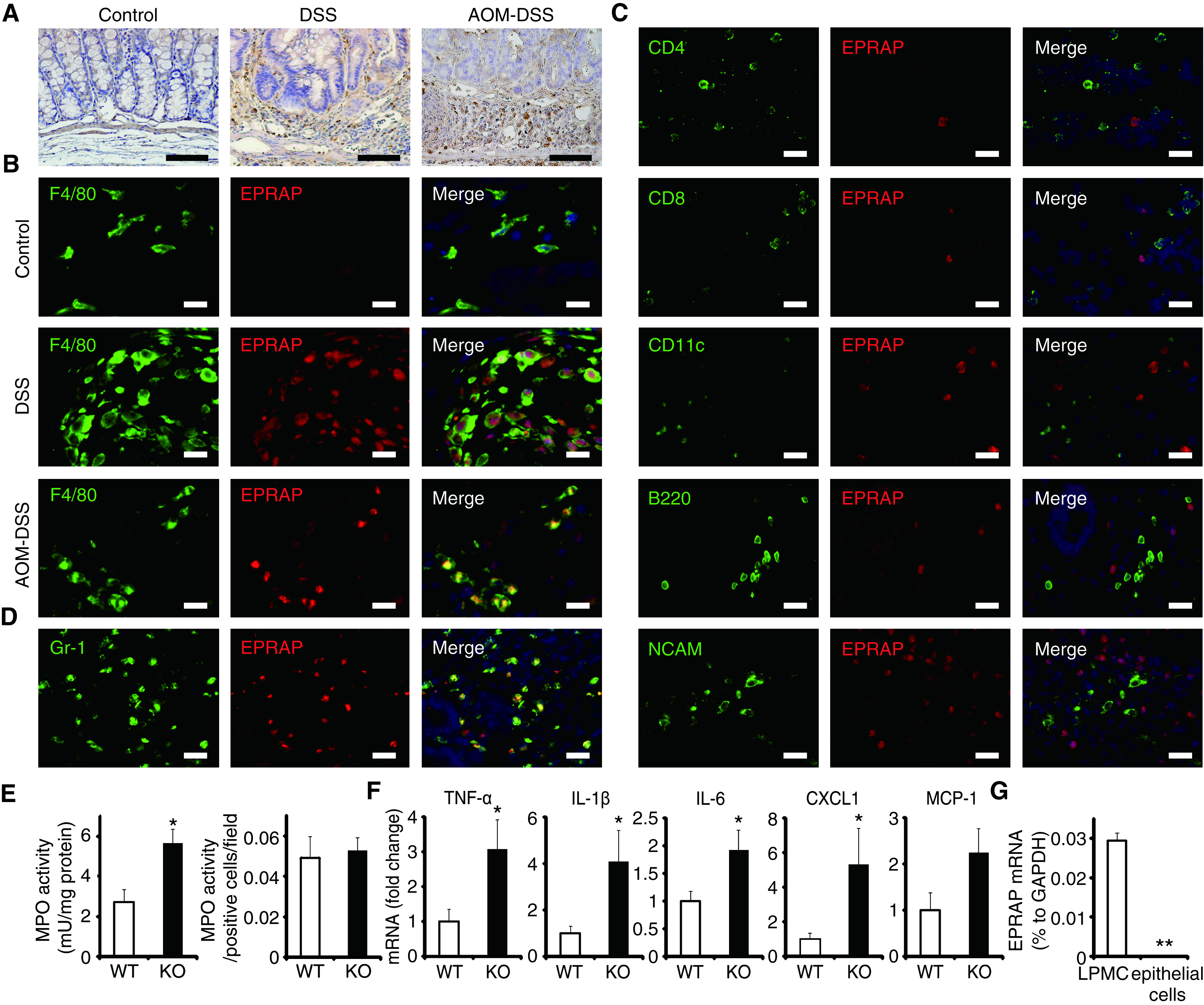

Supplement: S5 Fig — (A) Immunohistochemical detection of EPRAP-expressing cells. The colonic tissues were obtained from WT mice subjected to no treatment (drug-free water; normal colon), DSS treatment, or AOM/DSS treatment. Scale bars: 100 μm. (B) Double–color immunofluorescence staining of rectal sections from control-, DSS-, or AOM/DSS-treated WT mice was performed with a combination of anti-EPRAP (red) and anti-F4/80 (green) antibodies. Scale bars: 20 μm. (C) Double–color immunofluorescence staining of rectal sections from DSS-treated WT mice was performed using a combination of anti-EPRAP (red) and anti-CD4, anti-CD8, anti-CD11c, anti-B220, or anti-NCAM (green) antibodies. Scale bars: 20 μm. (D) Double–color immunofluorescence staining of rectal sections from DSS-treated WT mice was performed using a combination of anti-EPRAP (red) and anti–Gr-1 (green) antibodies. Scale bars: 20 μm. (E) MPO activity was measured in protein extracts from colonic tissues from DSS-treated WT and EPRAP-deficient (KO) mice (n = 6 [WT]; n = 4 [KO]). MPO activity (left) and MPO activity compensated by the numbers of Gr-1–positive cells infiltrated in colonic tissues per high-power field (Fig 1G) (right). (F) The mRNA levels of TNF-α, IL-1β, CXCL1, and MCP-1 in colonic stromal macrophages of DSS-treated WT and EPRAP-deficient (KO) mice (n = 7 [WT]; n = 5 [KO]). (G) The EPRAP mRNA levels of lamina propria macrophages (LPMC) and epithelial cells isolated from the colonic tissues of WT mice with DSS treatment. Data represent fold induction of mRNA expression compared with WT. *P < 0.05, **P < 0.01 vs. WT mice. (TIF) [file pgen.1005542.s005.tif]

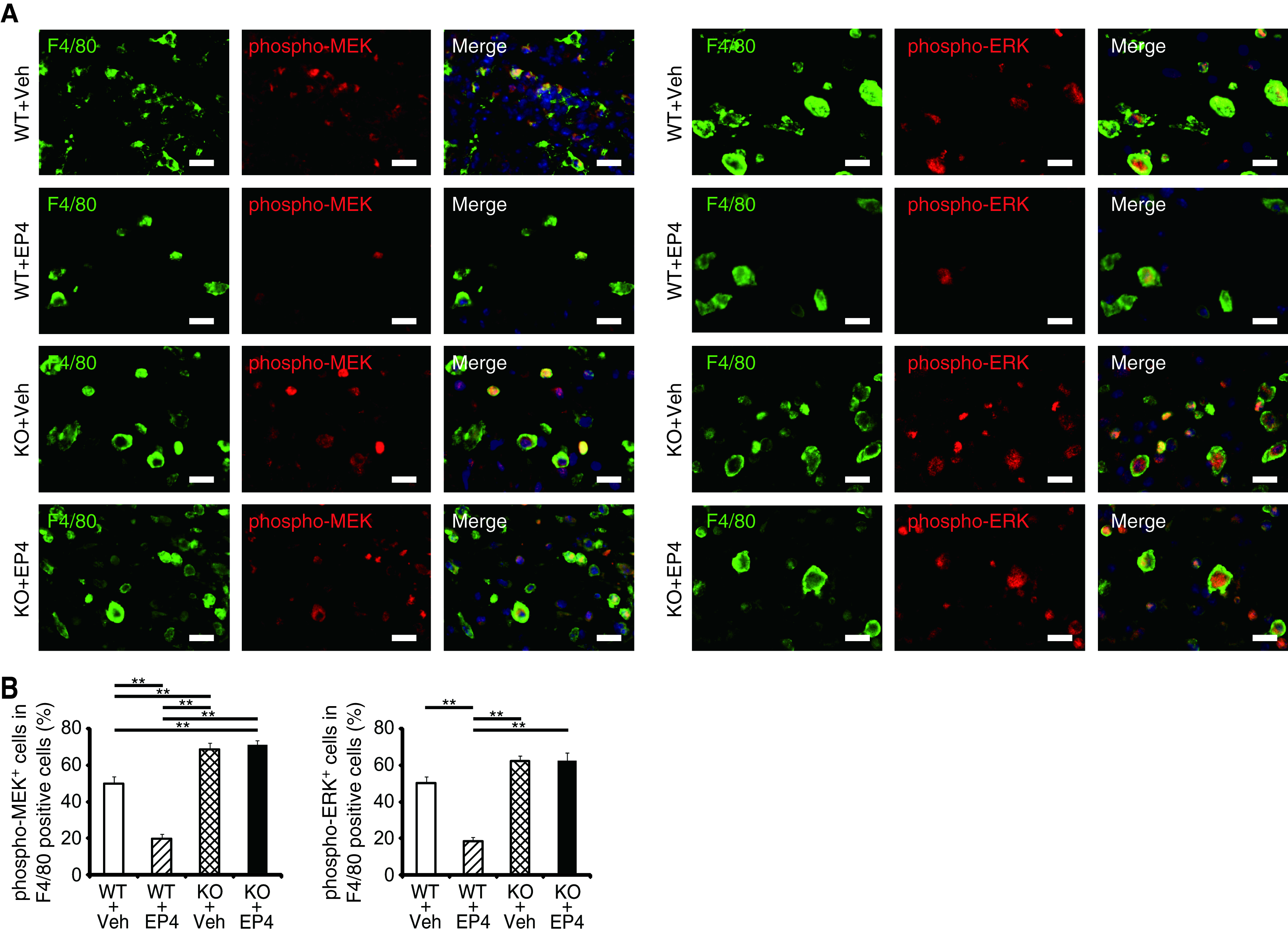

Supplement: S6 Fig — (A) Double-color immunofluorescence staining of rectal sections from DSS-treated WT and EPRAP deficient mice with or without EP4 agonist treatment (as described in the Fig 3 legend) was performed with anti-F4/80 (green) and anti–phospho-MEK (red) (left), anti–phospho-ERK (red) (right). Scale bars: 20 μm. (B) The percentages of phospho-MEK (left) or phospho-ERK (right) positive cells in F4/80 positive macrophages (n = 5 each). (TIF) [file pgen.1005542.s006.tif]

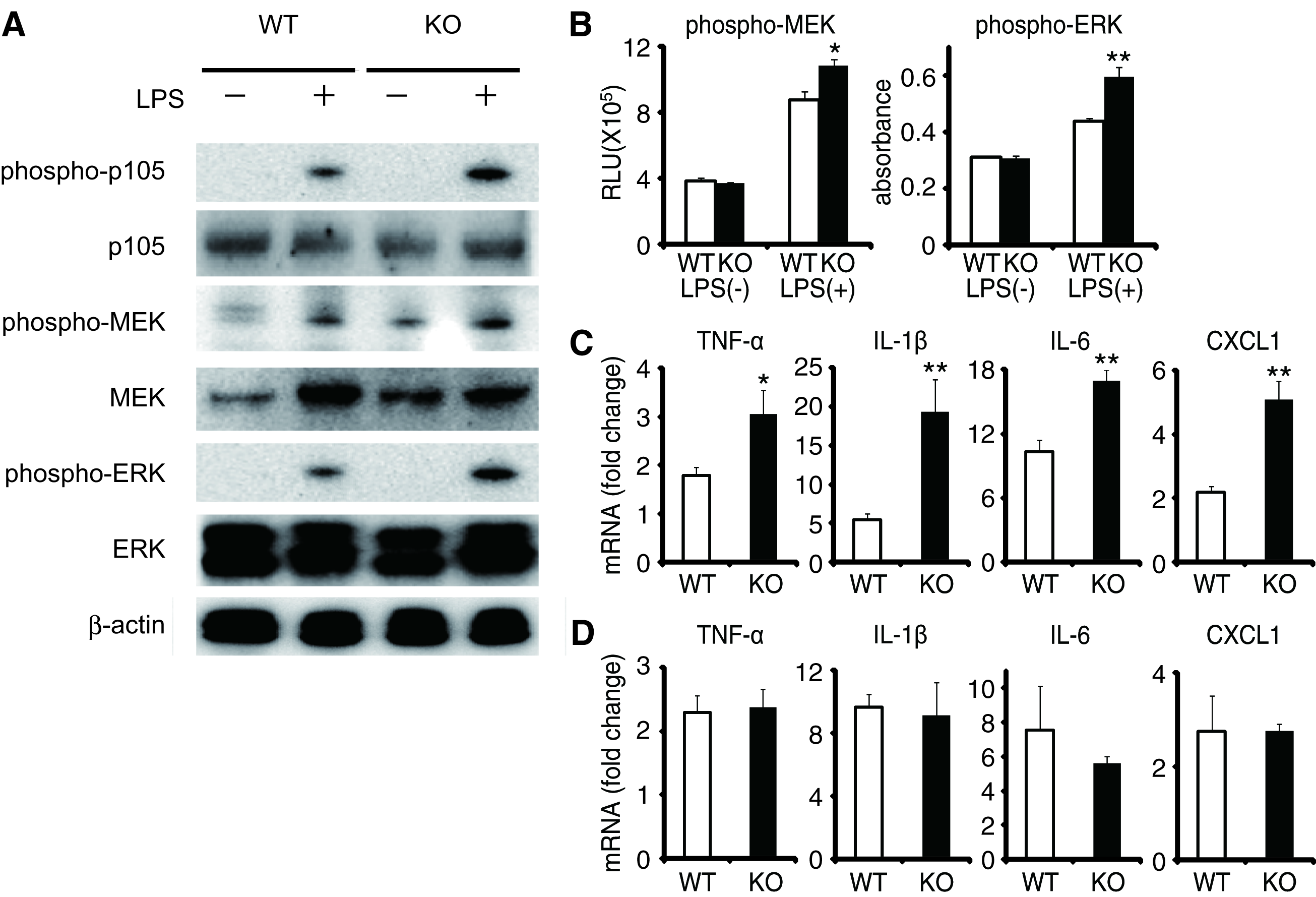

Supplement: S7 Fig — Peritoneal macrophages were isolated from 10–12-week-old of WT or KO mice. Cells were incubated with LPS or vehicle for one hour, followed by whole cell extraction. (A) Immunoblot analyses were performed to examine the levels of phosphorylated forms of p105, MEK and ERK. Figures are representative of three independent experiments. (B) The expression levels of phosphorylated forms of MEK and ERK were examined (n = 4 each). (C) The mRNA levels of TNF-α, IL-1β, IL-6, and CXCL1 in peritoneal macrophages of WT or KO mice (n = 7–9 each). (D) The mRNA levels of TNF-α, IL-1β, IL-6, and CXCL1 in peritoneal macrophages of WT or KO mice pre-treated with MEK inhibitor (U0126) (n = 6 each). Data represent fold induction of mRNA expression of LPS-treated cells compared with that of vehicle-treated cells. *P < 0.05, **P < 0.01 vs. WT mice. (TIF) [file pgen.1005542.s007.tif]

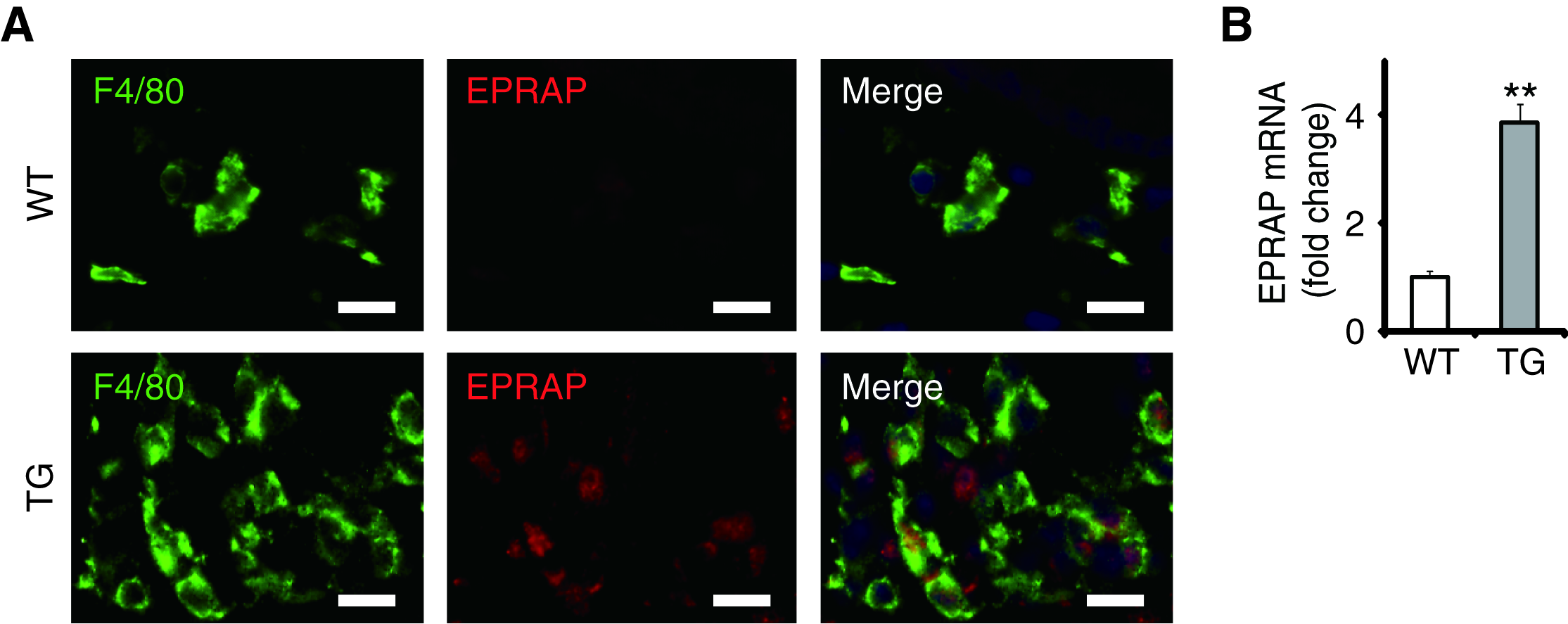

Supplement: S8 Fig — (A) Double–color immunofluorescence staining of rectal sections from WT and CD68–mEPRAP transgenic (TG) mice was performed with a combination of anti-EPRAP (red) and anti-F4/80 (green) antibodies. (B) The mRNA levels of EPRAP in CD68-positive bone marrow cells (n = 4 each). Data represent fold induction of mRNA expression compared with WT. **P < 0.01 vs. WT mice. (TIF) [file pgen.1005542.s008.tif]

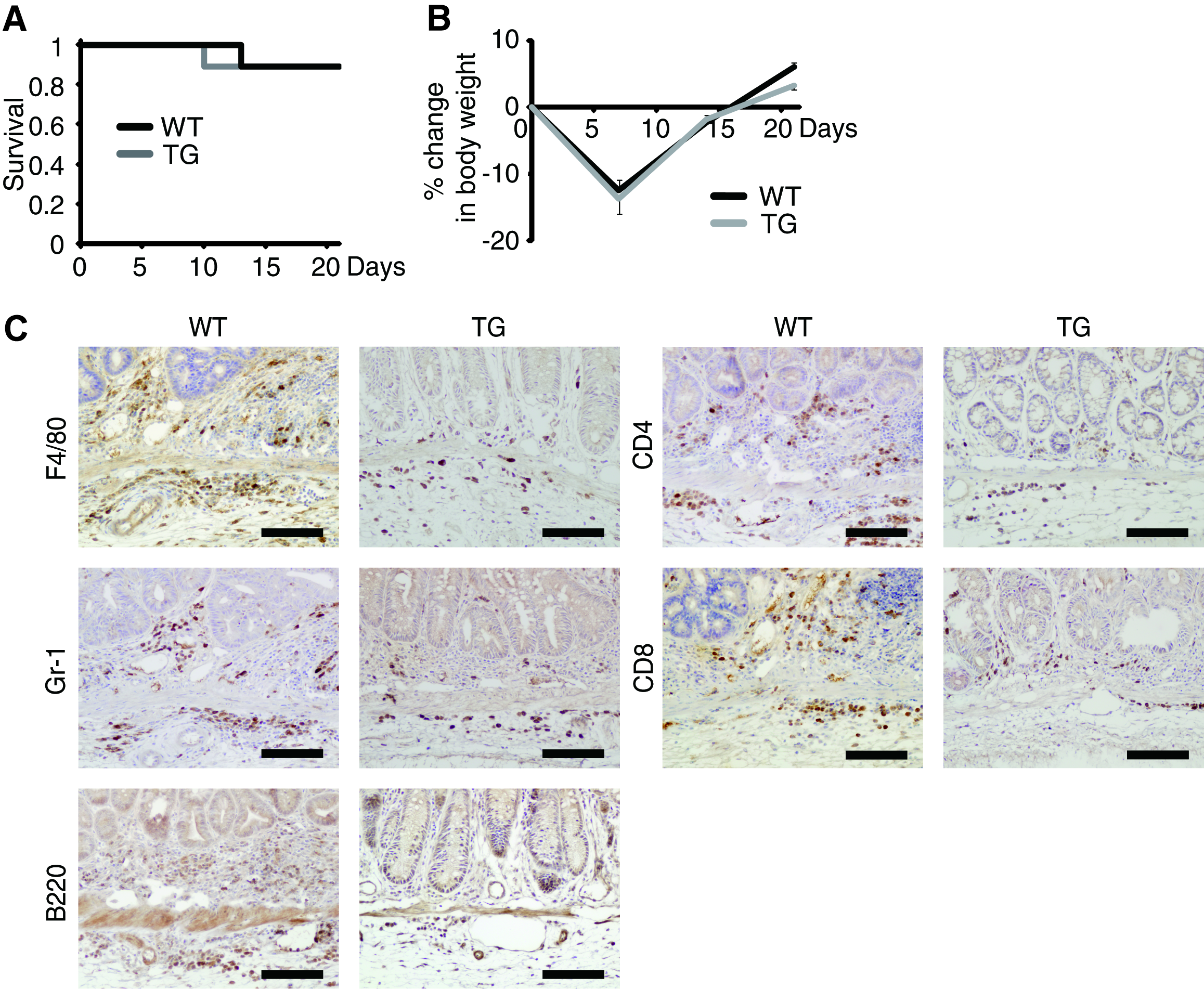

Supplement: S9 Fig — (A) Survival curves during the DSS treatment. WT and TG mice had no significant difference in mortality (n = 18 [WT]; n = 9 [TG]). (B) Percent changes in body weight (n = 16 [WT]; n = 8 [TG]). WT and TG mice had no significant difference. (C) Immunohistochemical staining to detect F4/80, Gr-1, B220, CD4, and CD8 in rectal sections of DSS-treated WT and TG mice. (TIF) [file pgen.1005542.s009.tif]

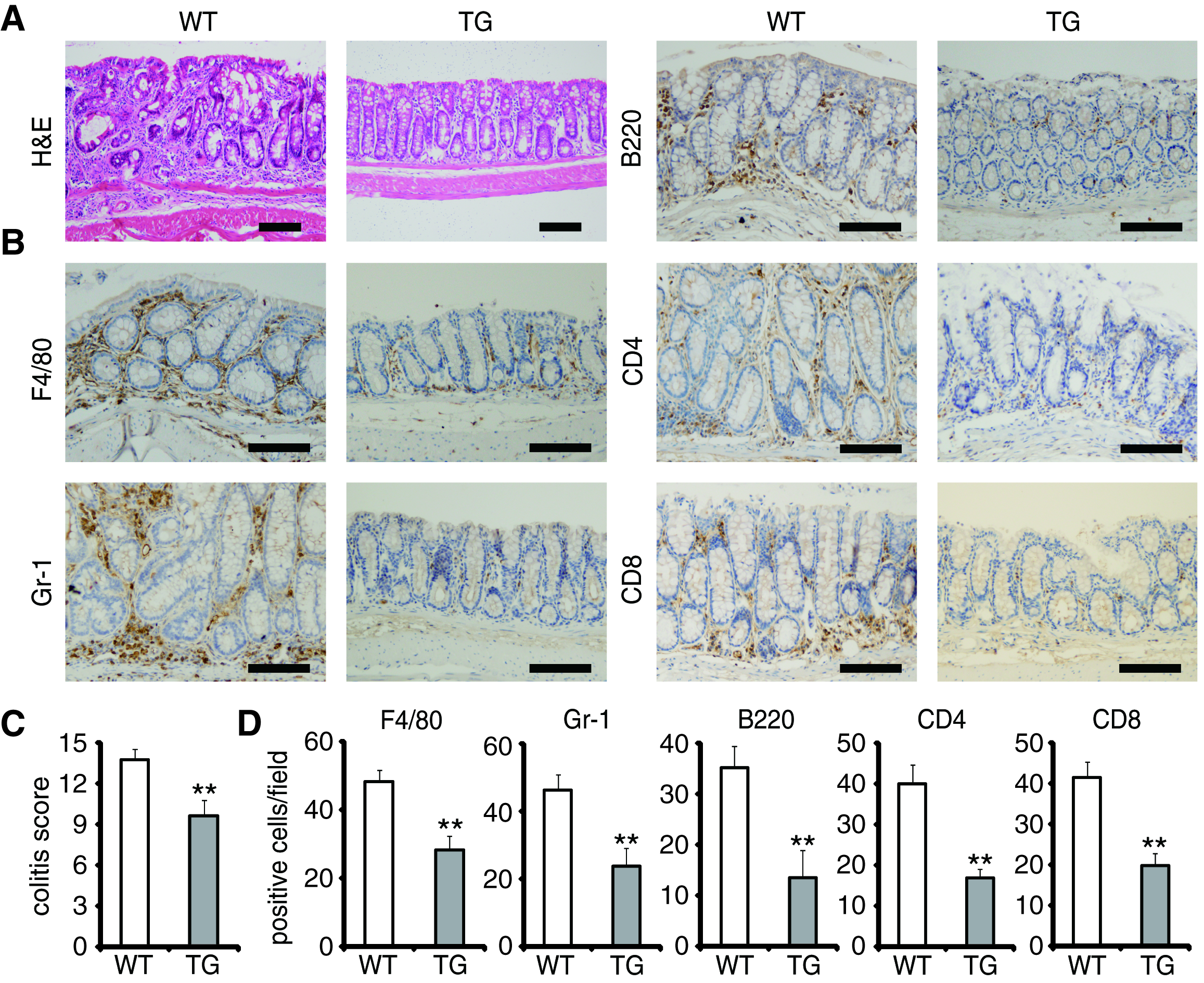

Supplement: S10 Fig — (A) H & E staining in rectal sections (non-polyp lesion) of AOM/DSS-treated WT and TG mice. (B) Immunohistochemical staining to detect F4/80, Gr-1, B220, CD4, and CD8 in rectal sections (non-polyp lesion) of AOM/DSS-treated WT and TG mice. (C) Histological colitis score in rectal sections (non-polyp lesion) of AOM/DSS-treated WT and KO mice (n = 9 [WT]; n = 8 [TG]). (D) The numbers of F4/80-, Gr-1–, B220-, CD4-, and CD8-positive cells infiltrated in colonic tissues per high-power field (400× magnification) in rectal sections (non-polyp lesion) of AOM/DSS-treated WT and TG mice (n = 9 [WT]; n = 8 [TG]). All values represent means ± SEM. **P < 0.01 vs. WT mice. Scale bars: 100 μm. (TIF) [file pgen.1005542.s010.tif]

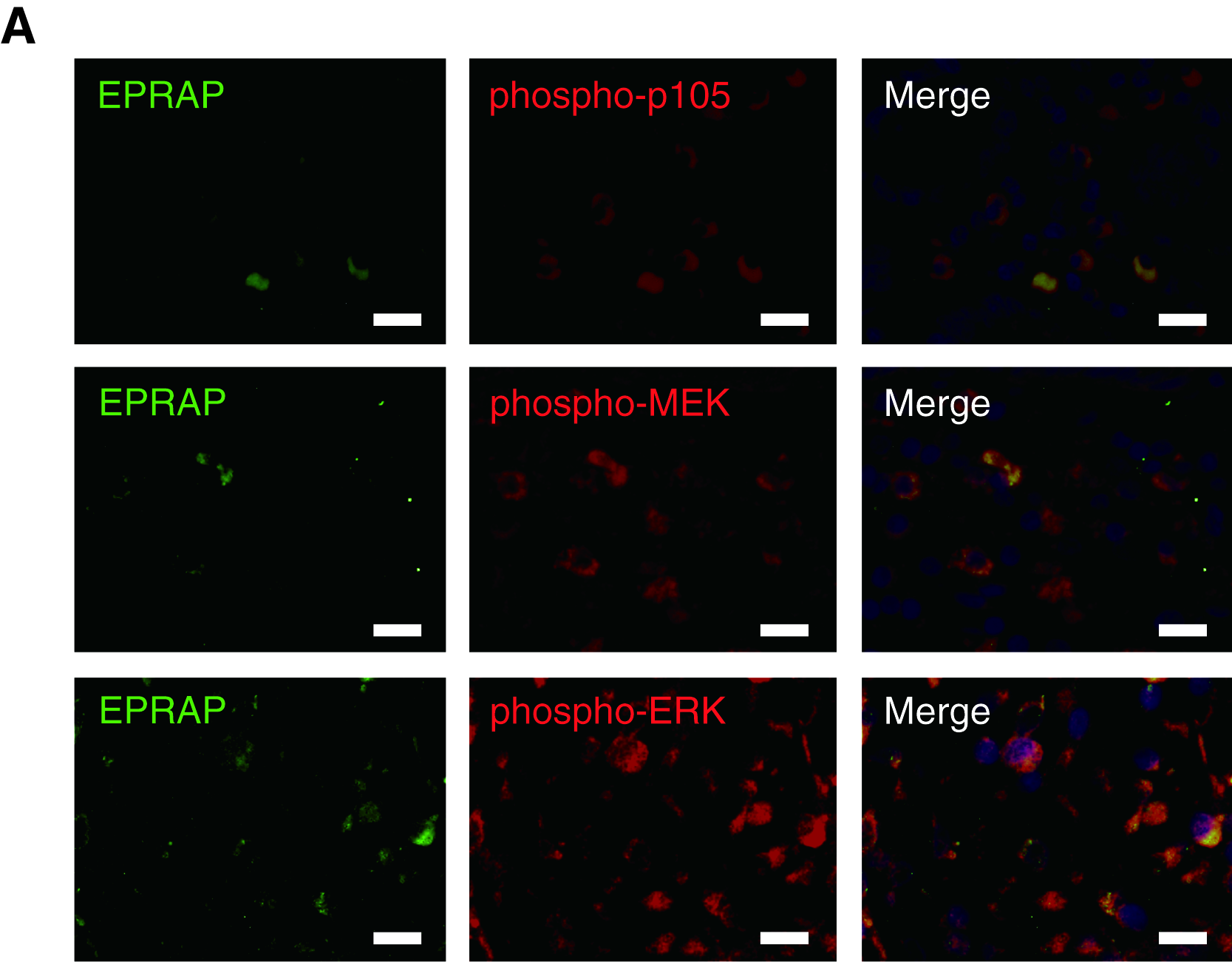

Supplement: S11 Fig — (A) Double–color immunofluorescence staining of colonic sections of UC patients was performed with a combination of anti-EPRAP (green) and anti–phospho-p105 (red, top), anti–phospho-MEK (red, middle), or anti–phospho-ERK (red, bottom) antibodies. Scale bars: 20 μm. (TIF) [file pgen.1005542.s011.tif]
