# Supplementary material for: EP4 Receptor–Associated Protein in Macrophages Ameliorates Colitis and Colitis-Associated Tumorigenesis
Source: PLoS Genet. 2015 Oct 6;11(10):e1005542. doi: 10.1371/journal.pgen.1005542 (PMC4595503; doi:10.1371/journal.pgen.1005542)
Supplement: S1 Table — (DOCX) [file pgen.1005542.s012.docx]

# S1 Table.

Classification of ulcerative colitis by severity

|  | Mild (n=2) | Moderate (n=20) | Severe (n=16) |
| --- | --- | --- | --- |
| (1) Frequency of defection | 4 times or less | Intermediate between severe and mild | 6 times or more |
| (2) Bloody stool | (+) to (-) |  | (+++) |
| (3) Fever | Absent |  | 37.5°C or higher |
| (4) Tachycardia | Absent |  | 90/min or more |
| (5) Anemia | Absen |  | Hb 10 g/dL or less |
| (6) Erythrocyte Sedimentation Rate | Normal |  | 30 mm/hour or more |

Notes)

- Rated as “severe” when criteria (1), (2) and one of the systemic symptoms (3) or (4) are satisfied, and at least 4 of the 6 criteria are satisfied.
- Rated as “mild” when all of the 6 criteria are satisfied.
