# Supplementary material for: EP4 Receptor–Associated Protein in Macrophages Ameliorates Colitis and Colitis-Associated Tumorigenesis
Source: PLoS Genet. 2015 Oct 6;11(10):e1005542. doi: 10.1371/journal.pgen.1005542 (PMC4595503; doi:10.1371/journal.pgen.1005542)
Supplement: S2 Table — (DOCX) [file pgen.1005542.s013.docx]

# S2 Table.

Primers used in this study

Target gene Orientation Sequence

EPRAP forward 5’-CCTCGCCCAGTATTCTTCGAG-3’

reverse (WT allele) 5’-AGCTCCTCCAGCTCCTCACG-3’

reverse (KO allele) 5’-TGTCACGTCCTGCACGACGC-3’

CD68–mEPRAP forward 5’-GGCAAAGGGGATTGGATTGAG-3’

reverse 5’-TACGGTTCTGCTTCTTCTGGGG-3’

mouse GAPDH forward 5’- AGGTCGGTGTGAACGGATTTG-3’

reverse 5’- TGTAGACCATGTAGTTGAGGTCA-3’

mouse EPRAP forward 5’-GAAGAAGCAGAACCGTATGAGAG-3’

reverse 5’-GGCTCCAAGCAGATCCCTTT-3’

mouse TNF-α forward 5’-CCCTCACACTCAGATCATCTTCT-3’

reverse 5’-GCTACGACGTGGGCTACAG-3’

mouse IL-1β forward 5’-GCAACTGTTCCTGAACTCAACT-3’

reverse 5’-ATCTTTTGGGGTCCGTCAACT-3’

mouse IL-6 forward 5’-TAGTCCTTCCTACCCCAATTTCC-3’

reverse 5’-TTGGTCCTTAGCCACTCCTTC-3’

mouse CXCL-1 forward 5’-CTGGGATTCACCTCAAGAACATC-3’

reverse 5’-CAGGGTCAAGGCAAGCCTC-3’

mouse MCP-1 forward 5’-TTAAAAACCTGGATCGGAACCAA-3’

reverse 5’-GCATTAGCTTCAGATTTACGGGT-3’

mouse EP4 forward 5’-CTTGTTGGTAAGCCCGGTGA-3’

reverse 5’-CCGACAGACCGAAGAAAAGTAG-3’

mouse iNOS forward 5’-GTTCTCAGCCCAACAATACAAGA-3’

reverse 5’-GTGGACGGGTCGATGTCAC-3’

mouse CXCL10 forward 5’-CCAAGTGCTGCCGTCATTTTC-3’

reverse 5’-GGCTCGCAGGGATGATTTCAA-3’
